# A phase 1b single-arm trial of intratumoral oncolytic virus V937 in combination with pembrolizumab in patients with advanced melanoma: results from the CAPRA study

**DOI:** 10.1007/s00262-022-03314-1

**Published:** 2022-11-29

**Authors:** Ann W. Silk, Steven J. O’Day, Howard L. Kaufman, Jennifer Bryan, Jacqueline T. Norrell, Casey Imbergamo, Daniella Portal, Edwin Zambrano-Acosta, Marisa Palmeri, Seymour Fein, Cai Wu, Leslie Guerreiro, Daniel Medina, Praveen K. Bommareddy, Andrew Zloza, Bernard A. Fox, Carmen Ballesteros-Merino, Yixin Ren, Darren Shafren, Mark Grose, Joshua A. Vieth, Janice M. Mehnert

**Affiliations:** 1 Rutgers Cancer Institute of New Jersey, New Brunswick, NJ USA; 2 Rutgers Robert Wood Johnson Medical School, New Brunswick, NJ USA; 3 Dana-Farber Cancer Institute, Boston, MA USA; 4 John Wayne Cancer Institute, Santa Monica, CA USA; 5 Department of Surgery, Massachusetts General Hospital, Boston, MA USA; 6 Virginia Mason Cancer Institute, Seattle, WA USA; 7 CNF Pharma, LLC, New City, NY USA; 8 Merck & Co., Inc, Rahway, NJ USA; 9 Rutgers Graduate School of Biomedical Sciences, New Brunswick, NJ USA; 10 Division of Hematology, Oncology, and Cell Therapy, Department of Internal Medicine, Rush University Cancer Center, Rush University Medical Center, Chicago, IL USA; 11 Earle A. Chiles Research Institute in the Providence Cancer Institute, Portland, OR USA; 12 ImmvirX Pty Ltd, New Lambton Heights, NSW Australia; 13 Viralytics Limited, a Wholly Owned Subsidiary of Merck & Co., Inc., Rahway, NJ USA; 14 Melanoma and Cutaneous Oncology, Laura and Isaac Perlmutter Cancer Center at NYU, New York University Langone Medical Center, 522 First Ave, SML1304, New York, NY 10016 USA; 15 Present Address: Rutgers New Jersey Medical School, Newark, NJ USA; 16 Present Address: JDRF International, New York, NY USA

**Keywords:** Clinical trial, Oncolytic virus, V937, Pembrolizumab, Melanoma

## Abstract

**Background:**

CAPRA (NCT02565992) evaluated Coxsackievirus A21 (V937) + pembrolizumab for metastatic/unresectable stage IIIB–IV melanoma.

**Methods:**

Patients received intratumoral V937 on days 1, 3, 5, and 8 (then every 3 weeks [Q3W]) and intravenous pembrolizumab 2 mg/kg Q3W from day 8. Primary endpoint was safety.

**Results:**

Median time from first dose to data cutoff was 32.0 months. No dose-limiting toxicities occurred; 14% (5/36) of patients experienced grade 3‒5 treatment-related adverse events. Objective response rate was 47% (complete response, 22%). Among 17 responders, 14 (82%) had responses ≥ 6 months. Among 8 patients previously treated with immunotherapy, 3 responded (1 complete, 2 partial). Responses were associated with increased serum CXCL10 and CCL22, suggesting viral replication contributes to antitumor immunity. For responders versus nonresponders, there was no difference in baseline tumor PD-L1 expression, *ICAM1* expression, or CD3^+^ infiltrates. Surprisingly, the baseline cell density of CD3^+^CD8^−^ T cells in the tumor microenvironment was significantly lower in responders compared with nonresponders (*P* = 0.0179).

**Conclusions:**

These findings suggest responses to this combination may be seen even in patients without a typical “immune-active” microenvironment.

**Trial registration number:**

NCT02565992.

**Supplementary Information:**

The online version contains supplementary material available at 10.1007/s00262-022-03314-1.

## Introduction

Oncolytic viruses represent a novel therapeutic modality for the treatment of cancer, and several have been or are currently being investigated as potential immunotherapies in patients with various types of cancers [1]. Oncolytic viruses are thought to mediate antitumor activity via two distinct mechanisms: direct cancer cell lysis and activation of a systemic antitumor immune response [2]. Talimogene laherparepvec, a genetically modified herpes simplex virus 1, was the first oncolytic virus to gain regulatory approval; it is indicated for the local treatment of unresectable lesions in patients with melanoma that recurred after initial surgery [3]. Coxsackievirus is an RNA virus that is associated with mild, common cold–like upper respiratory symptoms [4]. Coxsackievirus A21 (previously referred to as CVA21 or Cavatak; now referred to as V937) is a bioselected genetically unmodified strain that is oncolytic [5]. It enters cells via binding to intracellular adhesion molecule 1 (ICAM-1) and decay-accelerating factor (DAF) receptors [6], both of which are highly expressed on the surface of melanoma cells [7, 8].

In preclinical studies, V937 demonstrated rapid oncolysis of in vitro melanoma cell cultures and in vivo melanoma xenografts in immunodeficient mice [7, 8]. Notably, antitumor activity was observed at sites distant from the site of intratumoral administration [7, 8]. In the phase 2 CAvatak in Late-stage Melanoma (CALM) study [5], 57 patients with stage IIIC to IVM1c melanoma were treated with multiple doses of intratumoral V937. The 6-month progression-free survival (PFS) rate, the primary endpoint, and objective response rate (ORR) were both 38.6%, and 21.1% of patients achieved a durable complete or partial response lasting ≥ 6 months. Regression of melanoma was seen in injected lesions as well as noninjected lesions, consistent with induction of a systemic antitumor immune response. Treatment was well tolerated in the study, with no patients experiencing treatment-related adverse events (AEs) of grade ≥ 3.

Pharmacodynamic effects of oncolytic viruses in the tumor microenvironment, including increased interferon production, CD8^+^ T cells, and programmed death ligand 1 (PD-L1) expression [9] and reduced suppressor T-cell populations [10], suggest the potential for improved responses when combined with immune checkpoint inhibitors such as those targeting programmed death 1 (PD-1)/PD-L1 or cytotoxic T-lymphocyte antigen 4 (CTLA-4). Preliminary evidence from the phase 1b Melanoma Intra-Tumoral Cavatak and Ipilimumab (MITCI) study showed that the combination of intratumoral V937 and the anti–CTLA-4 monoclonal antibody ipilimumab was not associated with any dose-limiting toxicities (DLTs) in 23 patients with stage IIIC to IVM1c melanoma [11]. An ORR of 50% by investigator assessment was reported among the 18 patients evaluated for response, which suggests at least an additive effect with the combination. Similar to the V937 monotherapy experience, responses were seen in both injected and noninjected lesions [11].

The anti–PD-1 monoclonal antibody pembrolizumab is a standard-of-care therapy for patients with advanced melanoma. In the phase 3 KEYNOTE-006 study, which included 834 patients with metastatic melanoma (65% were stage IVM1c), pembrolizumab demonstrated superiority over ipilimumab with respect to the dual primary endpoints of PFS and overall survival (OS), with less high-grade toxicity [12]. The benefit of pembrolizumab was maintained over 5 years of follow-up [13]. The phase 1b CAvatak and PembRolizumab in Advanced melanoma (CAPRA) study (Protocol VLA-011; NCT02565992) evaluated the safety, efficacy, and correlative biomarker results associated with the combination of intratumoral V937 and intravenous pembrolizumab in patients with advanced melanoma.

## Methods

### Study design and patients

CAPRA was a phase 1b, multicenter, open-label, single-arm study. Eligible patients were ≥ 18 years old and had histologically confirmed metastatic or unresectable stage IIIB to IV melanoma, according to the American Joint Committee on Cancer 7th edition [14, 15]. Patients also had ≥ 1 cutaneous or subcutaneous tumor or palpable lymph node amenable to intratumoral injection; an Eastern Cooperative Oncology Group (ECOG) performance status of 0 or 1; and adequate hematologic, hepatic, and renal function. Prior T-cell checkpoint antibody therapy in the adjuvant or the metastatic setting was permitted. Use of chemotherapy, radiation therapy, hormonal treatment, or immunotherapy within 28 days before initiation of study treatment or previous receipt of V937 was prohibited. A complete list of the inclusion and exclusion criteria for the study can be found in the Supplementary Material (see Protocol). The protocol was approved by an appropriate institutional review board or independent ethics committee at each center, and the study was conducted in accordance with local laws, Good Clinical Practice guidelines, and the Declaration of Helsinki. All patients provided written informed consent.

### Treatment

Patients were treated with intratumoral V937 on days 1, 3, 5, and 8 and then every 3 weeks for up to 19 sets of injections. At each injection visit, the maximum dose of V937 administered was 3 × 10^8^ 50% tissue culture infectious dose (TCID_50_; approximately 4.5 × 10^6^ TCID_50_ per kg for a 70-kg patient) in a maximum volume of 4.0 mL. The volume could be reduced below 4 mL, with a consequent reduction in dose, if too few lesions of sufficient size were available to inject. Multiple lesions were injected at each injection visit if possible. Lesions > 25 mm in diameter were injected first (2.0 mL volume), followed by lesions 15 to 25 mm in diameter (1.0 mL volume) and then lesions 5 to < 15 mm in diameter (0.5 mL volume). After the initial injection of V937, any injected lesion that decreased to < 5 mm in diameter was injected with 0.1 mL volume. Tumor diameter was measured using a ruler/calipers or calipers on ultrasound. After administration of V937, the injection site was wiped with sterile tissue, and an occlusive dressing was applied to completely cover the injection site.

Patients also received intravenous pembrolizumab (2 mg/kg solution) starting on day 8 and continuing every 3 weeks for up to 2 years or until complete response, disease progression, or intolerable toxicity, whichever occurred first. On days in which patients received both V937 and pembrolizumab, V937 was administered first.

### Assessments and endpoints

Adverse events and DLTs were assessed based on the National Cancer Institute Common Terminology Criteria for Adverse Events, version 4.03. AEs were assessed from the time of initiating study treatment through 30 days after cessation of study treatment. DLTs were defined as any grade ≥ 3 toxicity related or possibly related to V937 with onset on or before the day 92 visit, except for lymphopenia, which was not considered a DLT.

Disease status was assessed every 6 weeks by computed tomography or magnetic resonance imaging. Response and progression were evaluated using immune-related response criteria derived from modified World Health Organization criteria, as previously described [16, 17].

Optional melanoma tumor biopsy samples were obtained at screening by incisional, excisional, or punch biopsy; archival tissue could be used in place of a fresh biopsy sample. Tumors selected for biopsy could have been target or nontarget lesions; target lesions had to be > 20 mm in total size, and sample collection could not affect the longest diameter for purposes of tumor assessment. Biomarker analyses performed on these tumor samples included NanoString-based interrogation of gene expression with the PanCancer IO 360™ Panel (NanoString, Seattle, WA, USA) and multiplex IHC (Supplementary Table S1). In addition, Luminex cytokine analysis was performed on day 1 and day 29 paired serum samples with a 48-plex human Cytokine/Chemokine/Growth Factor Panel A (Millipore Sigma, Burlington, MA) analyzed on a Luminex 2000 analyzer (Luminex Corporation, Austin, TX). Serum V937 levels, based on polymerase chain reaction, were assessed during the first 2 years of treatment (at every study visit through week 46, at week 52, and every 12 weeks thereafter). Anti-V937 antibody titers were also evaluated during the first 2 years of treatment (at baseline, week 4, week 22, every 3 weeks through week 46, at week 52, and every 12 weeks thereafter).

The primary endpoints were the occurrence of AEs, serious AEs, and DLTs. Secondary endpoints were ORR, PFS, OS, and duration of response. Biomarkers were an exploratory endpoint.

### Statistical analyses

As described in detail in the Supplementary Material (see Protocol), a Simon’s 2-stage design was used for sample size justification for ORR. The historical response rate was assumed to be 28% [5]. With 45 patients, the design yielded a 1-sided *α* = 0.05 level of significance with 90% power to detect a difference when the true ORR was 50%. The total sample size was estimated to be 50 patients to account for those who may not complete the postbaseline lesion assessment. However, study enrollment was stopped early because of prioritization of sponsor resources.

Safety and efficacy analyses were performed in all patients who received treatment. Biomarker analyses, performed in all patients with data available, are exploratory; *P* values were based on Welch’s *t* test (unequal variance). For ORR analysis, 95% CIs were based on the exact method for binomial data. PFS, OS, and duration of response were analyzed using the Kaplan–Meier method.

## Results

### Patients

A total of 36 patients were enrolled between December 17, 2015 and November 4, 2019 (Supplementary Table S2). All patients received the combination of V937 and pembrolizumab. Of the 36 patients enrolled, 7 (19%) completed and 29 (81%) discontinued the study. Progressive disease was the most common reason for discontinuation (*n* = 20), followed by death (*n* = 2), physician decision (*n* = 2), and other (*n* = 5; Supplementary Figure S1). Median time from first dose to data cutoff date was 32.0 (range, 10.7–45.3) months.

Patients had a median age of 68.5 (range, 43–94) years and 27 (75%) were men (Table 1). More than half of patients (*n* = 21, 58%) had an ECOG performance status of 0. Twenty-three patients (64%) had stage IV disease and 13 patients (36%) had stage III disease. Previous therapies included immunotherapy (*n* = 8, 22%), adjuvant therapy (*n* = 3, 8%), and radiotherapy (*n* = 2, 6%). No patient had previously received chemotherapy or neoadjuvant therapy.

**Table 1:** Demographics and baseline characteristics

	*N* = 36
Median age (range), years	68.5 (43–94)
Male, *n* (%)	27 (75)
Race, *n* (%)	
White	33 (92)
American Indian or Alaska Native	1 (3)
Asian	1 (3)
Black or African American	1 (3)
ECOG PS, *n* (%)	
0	21 (58)
1	15 (42)
Metastatic tumor stage, *n* (%)	
M0	13 (36)
M1a	3 (8)
M1b	6 (17)
M1c	14 (39)
Previous cancer therapy,^a^ *n* (%)	11 (31)
Chemotherapy	0
Immunotherapy	8 (22)
Adjuvant therapy	3 (8)
Neoadjuvant therapy	0
Radiotherapy	2 (6)
Median tumor size (range),^b^ mm^2^	821 (79–13,030)

ECOG PS, European Cooperative Oncology Group performance status

^a^Patients may have received > 1 therapy

^b^Sum of product of perpendicular of target lesions

### Safety and tolerability

Adverse events occurring during the study are summarized in Table 2. No DLTs occurred. Treatment-related AEs occurred in 28 (78%) patients with 5 (14%) patients experiencing grade 3–5 treatment-related AEs. The most frequently occurring treatment-related AEs were rash (*n* = 14, 39%), fatigue (*n* = 12, 33%), pruritus (*n* = 6, 17%), diarrhea (*n* = 5, 14%), dry mouth (*n* = 5, 14%), and hypothyroidism (*n* = 4, 11%). Serious treatment-related AEs were experienced by 3 (8%) patients and included grade 3 keratoacanthoma, grade 3 autoimmune hepatitis, grade 3 autoimmune encephalitis, and grade 5 septic shock (the latter 2 events occurring in 1 patient), all of which were considered related to pembrolizumab by the investigator. There was 1 other grade 5 AE (cardiac failure), which was not considered treatment related by the investigator. Treatment-related AEs that led to treatment discontinuation were grade 3 increased hepatic enzymes and the grade 5 septic shock, the latter of which was related to steroids that had been administered for grade 3 autoimmune encephalitis.

**Table 2:** Safety and tolerability

	*N* = 36^a^	
	Any AE	Grade 3–5
AEs	34 (94)	13 (36)
Serious	11 (31)	8 (22)
Leading to discontinuation	3 (8)	3 (8)
Leading to death	2^b^ (6)	2^b^ (6)
Treatment-related AEs	28 (78)	5 (14)
Serious	3^c^ (8)	3^c^ (8)
Leading to discontinuation	2^d^ (6)	2^d^ (6)
Leading to death	1^b^ (3)	1^b^ (3)
Treatment-related AEs occurring in > 1 patient		
Rash	14 (39)	0
Fatigue	12 (33)	0
Pruritus	6 (17)	0
Diarrhea	5 (14)	0
Dry mouth	5 (14)	0
Hypothyroidism	4 (11)	0
Myalgia	3 (8)	0
Pyrexia	3 (8)	0
Vitiligo	3 (8)	0
Arthralgia	2 (6)	0
Decreased appetite	2 (6)	0
Dry skin	2 (6)	0
Injection site pain	2 (6)	0
Immune-mediated AEs (irrespective of relationship to treatment)^e^	8 (22)	4 (11)
Hypothyroidism	4 (11)	0
Adrenal insufficiency	1 (3)	0
Colitis	1 (3)	1 (3)
Encephalitis	1 (3)	1 (3)
Hepatitis	1 (3)	1 (3)
Hyperthyroidism	1 (3)	0
Pneumonitis	1 (3)	1 (3)

AE, adverse event

^a^Data are presented as *n* (%)

^b^Two patients died because of grade 5 AEs (1 from cardiac failure, 1 from septic shock); the septic shock was considered treatment related

^c^One patient had grade 3 autoimmune encephalitis and grade 5 septic shock, 1 had grade 3 keratoacanthoma, and 1 had grade 3 autoimmune hepatitis

^d^One patient had grade 3 increased hepatic enzyme, and 1 had grade 5 septic shock

^e^No infusion reactions were reported; no grade 4 or 5 immune-mediated AEs occurred

Immune-mediated AEs, regardless of relationship to treatment, occurred in 8 (22%) patients (Table 2). The most common immune-mediated AE was hypothyroidism (*n* = 4, 11%); no other immune-mediated AEs occurred in more than 1 patient. Four (11%) patients experienced grade 3 immune-mediated AEs including encephalitis, hepatitis, colitis, and pneumonitis (*n* = 1 for each). There were no grade 4 or 5 immune-mediated AEs. No infusion reactions occurred during the study.

### Efficacy

Efficacy results are summarized in Table 3. The best overall response based on immune-related response criteria was complete response in 8 (22%) patients and partial response in 9 (25%) patients, for an ORR of 47% (95% CI 30–65%). Among the 8 patients who previously received immune checkpoint inhibitor therapy, the ORR was 38%.

**Table 3:** Efficacy outcomes

	*N* = 36
Best overall response, *n* (%)	
CR	8 (22)
PR	9 (25)
SD	8 (22)
PD	10 (28)
No assessment	1 (3)
ORR (CR + PR), % (95% CI)	47 (30–65)
Duration of response	
Median (range), mo^a^	NR (1.4+ to 22.0+)
≥ 12 mo, %^a^	74
Progression-free survival	
Events, *n* (%)	20 (56)
Median (95% CI), mo^a^	11.9 (3.4–NR)
12-mo rate (95% CI), %^a^	45 (28–60)
Overall survival	
Events, *n* (%)	16 (44)
Median (95% CI), mo^a^	30.9 (20.3–40.5)
12-mo rate (95% CI), %^a^	85 (68–94)

+, no PD by the time of last disease assessment; CR, complete response; NR, not reached; ORR, objective response rate; PD, progressive disease; PR, partial response; SD, stable disease

Response and progression-free survival endpoints were with confirmation based on investigator assessment per immune-related response criteria

^a^Kaplan-Meier estimate

**Fig. 1:**
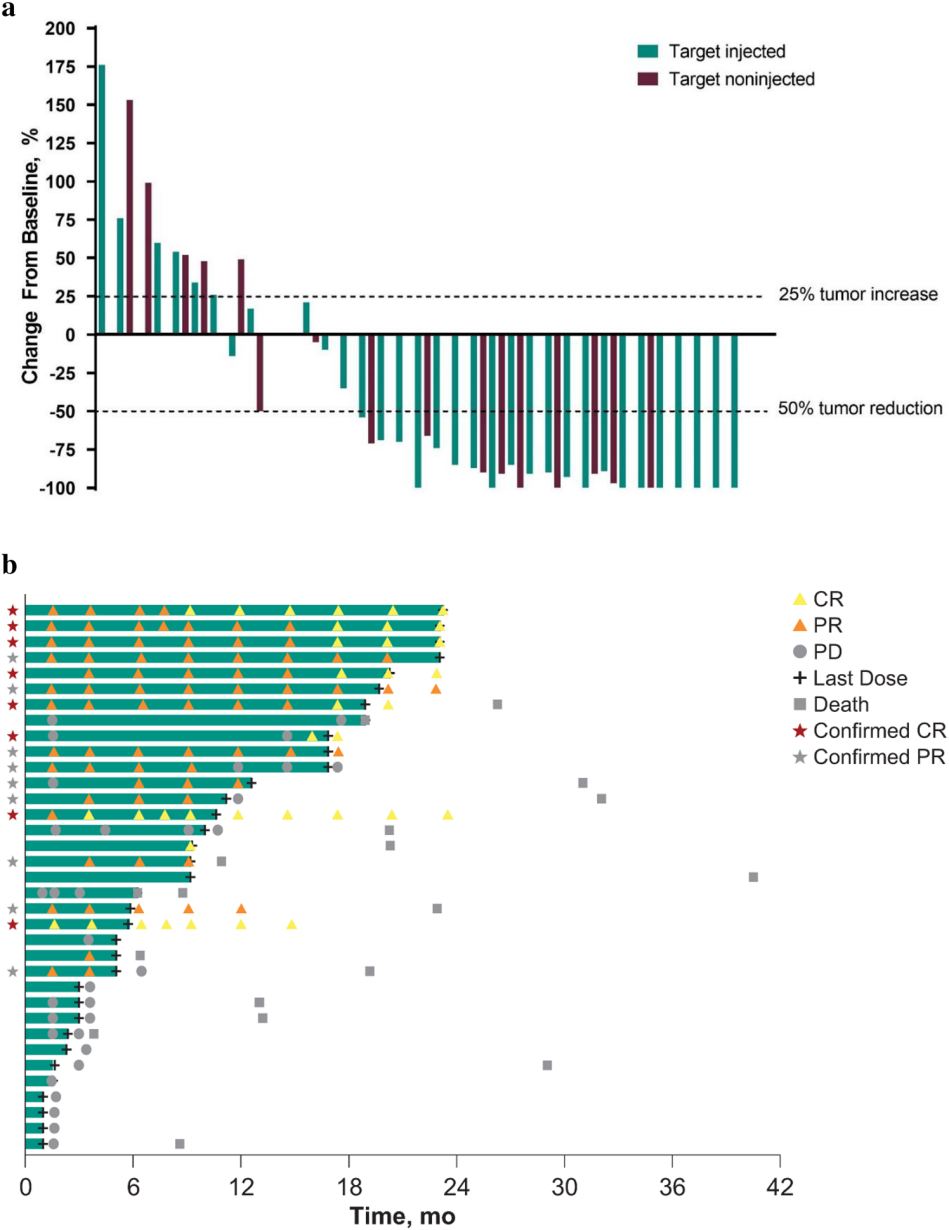
**a** Waterfall plot showing the best percentage change from baseline in size of target-injected (blue) and target-noninjected (maroon) lesions (all patients with ≥ 1 postbaseline assessment of target lesions). **b** Swimmer’s plot demonstrating treatment duration and response evaluation (investigator assessment per immune-related response criteria). CR, complete response; PD, progressive disease; PR, partial response

Among patients with ≥ 1 postbaseline assessment of target lesions, a ≥ 50% tumor volume reduction from baseline was observed in the size of target-injected lesions for 21 of 34 (62%) patients and target-noninjected lesions for 10 of 16 (63%) patients (Fig. 1a). Responses were largely concordant between injected and noninjected tumors. The duration of treatment and outcomes for each patient are shown in Fig. 1b. Among the 17 responders, the proportion with a complete or partial response lasting ≥ 6 months (i.e., a durable response) was 82% (95% CI 57–96%). The median time to response was 1.5 (range, 1.5–16.0) months, and the median duration of response was not reached. In Kaplan–Meier analysis, 74% of responders were estimated to have a response duration ≥ 12 months.

A total of 20 (56%) patients experienced a PFS event (disease progression or death; Table 3). Median PFS was 11.9 (95% CI 3.4–not reached) months. The PFS rates at 6 and 12 months were 60% (95% CI 42–74%) and 45% (95% CI 28–60%), respectively. A total of 16 (44%) patients died. Median OS was 30.9 (95% CI 20.3–40.5) months. The OS rate at 12 months was 85% (95% CI 68–94%).

### Biomarkers, viral load, and antibodies

**Fig. 2:**
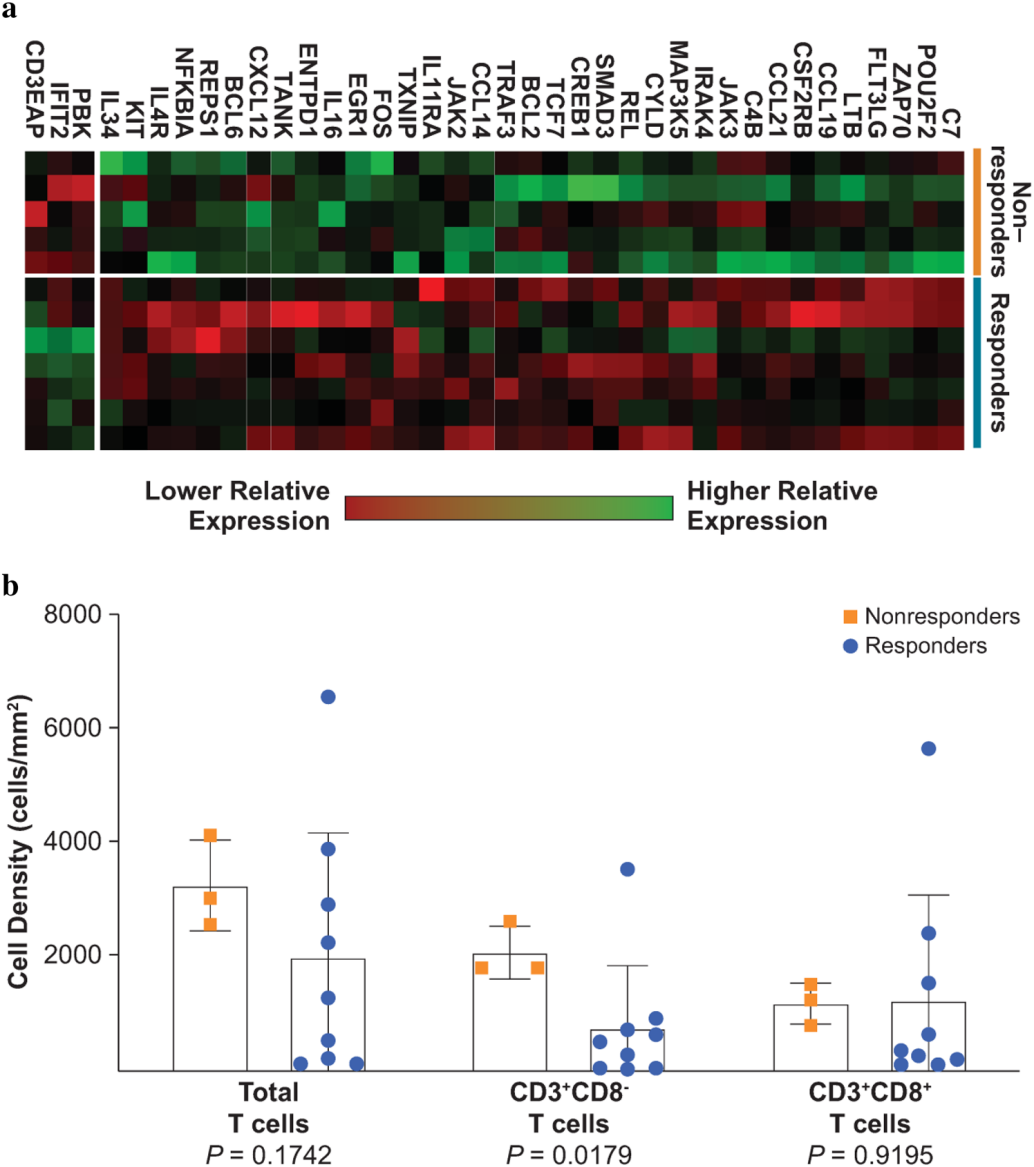
**a** PanCancer IO 360™ panel genes with differential expression in responders versus nonresponders in pretreatment tumor samples (all *P* < 0.05). **b** Multiplex immunohistochemistry analysis of T-cell populations in pretreatment tumor samples among responders and nonresponders

Baseline tumor samples from 12 patients were available and analyzed. NanoString analysis demonstrated significantly higher expression in pretreatment tumor samples from responders versus nonresponders of 3 genes (CD3e molecule, epsilon associated protein [*CD3EAP*], interferon-induced protein with tetratricopeptide repeats 2 [*IFIT2*], and PDZ-binding kinase [*PBK*]) and lower expression of 35 genes (all *P* < 0.05; Fig. 2a). No differences in gene expression were seen in the rest of the panel, including *ICAM1, DAF, PD-L1*, retinoic acid–inducible gene I (*RIG-I*), toll-like receptor 7 (*TLR7*), and toll-like receptor 8 (*TLR8*).

No differences were observed between responders and nonresponders in baseline cell density of total T cells or CD3^+^CD8^+^ T cells (both *P* > 0.05) by multiplex immunohistochemistry (IHC) analysis (Fig. 2b). Surprisingly, the baseline cell density of CD3^+^CD8^−^ T cells in the tumor microenvironment was lower in responders compared with nonresponders (*P* = 0.0179; Fig. 2b).

**Fig. 3:**
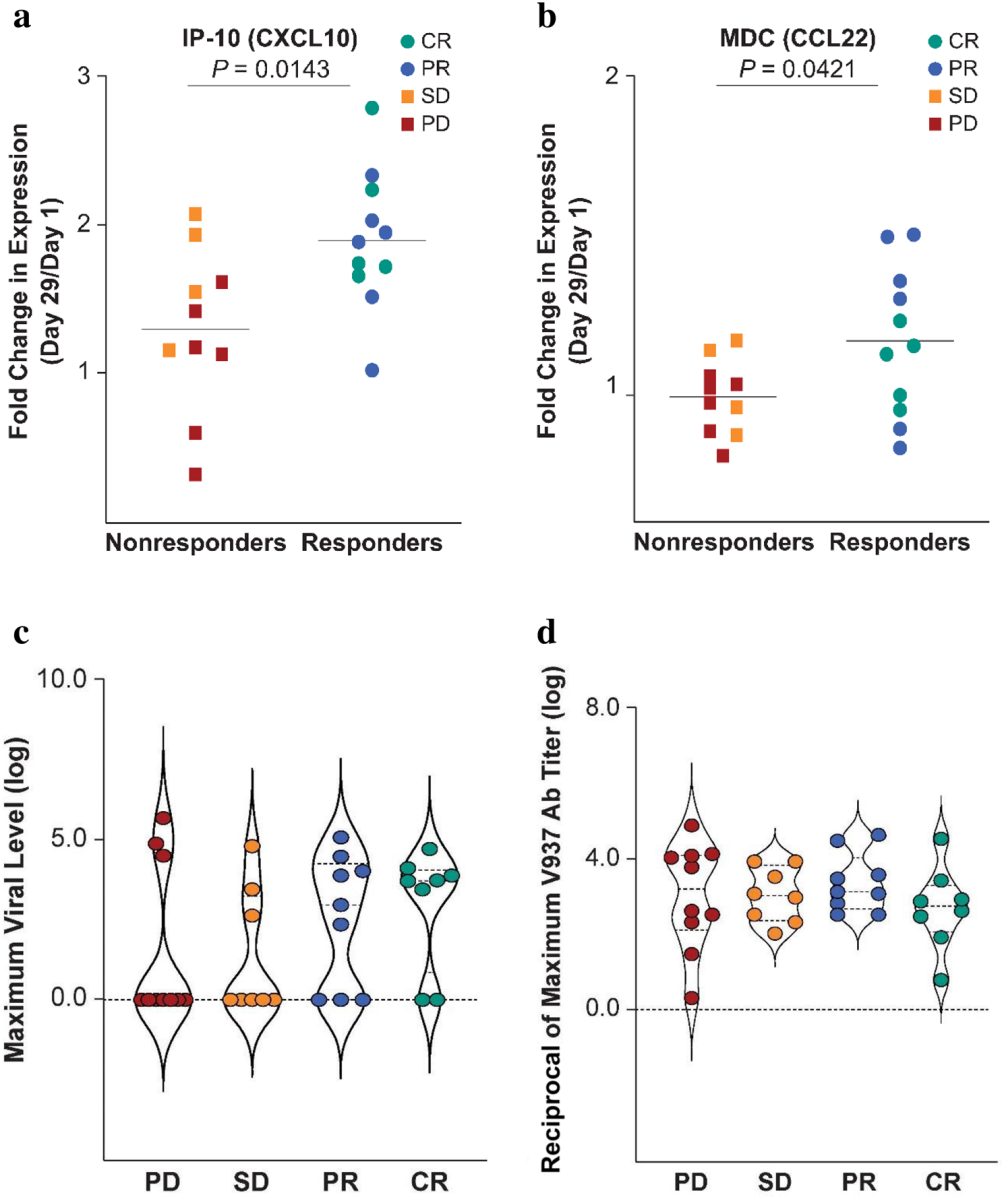
Increase in cytokine levels (day 29/day 1) from paired serum samples: **a** IP-10/CXCL-10 (*P* = 0.0143) and **b** MDC/CCL22 (*P* = 0.0421). Maximum circulating levels of **c** V937 during days 3–8 for each patient and **d** anti-V937 antibody by best overall response (investigator assessment per immune-related response criteria). In panels c and d, the dashed lines within the shapes represent the median and interquartile range. CR, complete response; PD, progressive disease; PR, partial response; SD, stable disease

Patients who responded to treatment exhibited significant increases in the expression of interferon-inducible protein 10 (IP-10/CXCL10) and macrophage-derived chemokine (MDC/CCL22) from day 1 to day 29 compared with nonresponders (*P* = 0.0143 and *P* = 0.0421, respectively; Fig. 3a, b). Maximum levels of circulating V937 during days 3–8 were numerically, but not significantly, higher in responders (detectable in 71% [12/17] of patients) than in nonresponders (detectable in 33% [6/18] of patients), whereas anti-V937 antibody levels were similar between responders and nonresponders (Fig. 3c, d).

## Discussion

In the phase 1b CAPRA study, we evaluated the combination of intratumoral V937 and pembrolizumab in patients with advanced melanoma and demonstrated a manageable safety profile and encouraging antitumor activity. No new safety signals were identified; the types of treatment-related AEs reported were as anticipated for each drug, given prior history with V937 and pembrolizumab, and were mostly of grade 1 or 2 severity. No myocarditis events occurred. The most common immune-mediated AE, regardless of relationship to treatment, was hypothyroidism both with combination therapy in CAPRA (11%) and with pembrolizumab monotherapy in a pooled analysis (9.1%) [18]. Hypothyroidism was managed in our study with corticosteroids and/or hormone replacement therapy and did not result in treatment discontinuation.

Notwithstanding the limitations of cross-study comparisons, based on immune-related response criteria, median PFS with the combination of V937 and pembrolizumab in CAPRA (11.9 months) was numerically greater than with V937 monotherapy in CALM (5.7 months) [5] or with pembrolizumab monotherapy (8.4 months) in an exploratory analysis from the KEYNOTE-006 study [13]. The ORRs, based on immune-related response criteria, were 47% in CAPRA, 38.6% in CALM [5], and 42% in KEYNOTE-006 [13]. The ORR in CAPRA was comparable to the preliminary rate of 50% achieved with the combination of V937 plus ipilimumab in MITCI [11]. The response rate of 47% in our study was similar to that reported from the phase 3 MASTERKEY-265 study of talimogene laherparepvec in combination with pembrolizumab (49%), although it is important to note that the patients in that study were naive to anti–PD-(L)1 therapy [19]. Another difference was that we administered V937 first and delayed pembrolizumab until day 8 after the fourth injection, whereas both drugs were administered starting on day 1 in the MASTERKEY-265 study.

Biomarkers were explored in CAPRA to gain insight into the possible mechanism for the systemic clinical responses. Surprisingly, responses were not associated with an increased baseline expression of viral entry proteins (ICAM-1 and DAF), PD-L1, or viral signaling proteins (RIG-I, TLR7, and TLR8), nor were they associated with an inflamed baseline tumor microenvironment. In fact, levels of infiltrating CD3^+^CD8^−^ T cells in pretreatment tumor samples were actually lower in responders compared with nonresponders. Although the reason for this finding is unknown, one hypothesis is that the pretreatment tumor samples of responders included fewer regulatory cells. We observed that V937 viral load was more often detectable in responders. Robust viral replication is a major determinant of effective oncolysis [8]. Another hypothesis to explain the finding that a lower number of tumor-infiltrating lymphocytes was associated with a higher rate of response is that fewer immune cells were immediately available in the tumor to combat the overwhelming coxsackievirus infection, which resulted in more robust viral replication locally and an increased circulating viral load. We also observed a significant increase in serum IP-10/CXCL10 in responding patients, and to a lesser extent, an increase in MDC/CCL22. CXCL10 is a marker of the severity of viral infection and facilitates recruitment of T cells, natural killer cells, macrophages, and dendritic cells [20]. Evidence suggests that CXCL10 is necessary for recruitment of antitumoral T cells into melanoma tumors [21]. Furthermore, CXCL10 signaling through the CXCR3 receptor promotes the migration of lymphocytes to dendritic cells, which was necessary for response to PD-1 blockade in a transplantable mouse model [22]. Clinically, there is evidence that high pretreatment CXCL9 and CXCL10 levels are correlated with response to anti–PD-(L)1 therapy in patients with non–small-cell lung cancer [23], and CXCL9 and CXCL10 increase in the first few months of treatment in patients with melanoma responding to PD-1 inhibitor therapy [22].

Given our findings above and the supportive data in the literature, we hypothesize that V937 viral infection leads interferon-gamma–mediated secretion of CXCL10 by multiple cell types in the tumor microenvironment, especially macrophages [24], which promotes response to anti–PD-1 therapy through improving local guidance of activated lymphocytes to the dendritic cells [22]. Quantitatively and qualitatively greater migration of T cells, including those recognizing V937 and tumor-associated antigens, furthered by increased expression of PD-L1 within the tumor microenvironment after treatment initiation, may also have contributed to efficacy. Unfortunately, we lack postbaseline biopsies, T-cell migration assays, T-cell phenotyping, or tetramer assays to help substantiate this hypothesis. Lastly, V937 is known to be able to induce innate immunity by cytokine-mediated bystander killing and natural killer cell killing in the cells of cancer patients [25], but innate assays were not explored in the current study.

In conclusion, the combination of intratumoral V937 plus pembrolizumab had favorable safety and efficacy in patients with advanced melanoma. The ORR was 47%, and there was a high rate of complete responses (22%) not typically seen with pembrolizumab monotherapy. Thus, the addition of V937 to pembrolizumab appeared to improve efficacy with limited impact on tolerability. Biomarker analysis showed that responses were not associated with an inflamed baseline tumor microenvironment, indicating that a pre-existing tumor immune infiltrate is not required for response to this combination. Markers of the intensity of the viral infection (CXCL10, CCL22) were associated with response, suggesting that the downstream effects of viral replication contributed to antitumor immunity and potentiated the activity of pembrolizumab. Further evaluation of the combination of V937 plus pembrolizumab for the neoadjuvant treatment of stage III melanoma is being assessed in an ongoing phase 1/2, randomized, open-label study (KEYMAKER-U02 substudy 02C; NCT04303169).

## Supplementary Information

Below is the link to the electronic supplementary material.

Supplementary file1 (DOCX 92 kb)

Supplementary file2 (PDF 1551 kb)

Supplementary file3 (EPS 2405 kb)

## Data Availability

Merck Sharp & Dohme LLC, a subsidiary of Merck & Co., Inc., Rahway, NJ, USA (MSD) is committed to providing qualified scientific researchers access to anonymized data and clinical study reports from the company’s clinical trials for the purpose of conducting legitimate scientific research. MSD is also obligated to protect the rights and privacy of trial participants and, as such, has a procedure in place for evaluating and fulfilling requests for sharing company clinical trial data with qualified external scientific researchers. The MSD data sharing website (available at: http://engagezone.msd.com/ds_documentation.php) outlines the process and requirements for submitting a data request. For this article, contact MSD at dataaccess@merck.com for access to the clinical data and Janice M. Mehnert at janice.mehnert@nyulangone.org for access to the biomarker data. Applications will be promptly assessed for completeness and policy compliance. Feasible requests will be reviewed by a committee of MSD subject matter experts to assess the scientific validity of the request and the qualifications of the requestors. In line with data privacy legislation, submitters of approved requests must enter into a standard data-sharing agreement with MSD before data access is granted. Data will be made available for request after product approval in the US and EU or after product development is discontinued. There are circumstances that may prevent MSD from sharing requested data, including country- or region-specific regulations. If the request is declined, it will be communicated to the investigator. Access to genetic or exploratory biomarker data requires a detailed, hypothesis-driven statistical analysis plan that is collaboratively developed by the requestor and MSD subject matter experts; after approval of the statistical analysis plan and execution of a data-sharing agreement, MSD will either perform the proposed analyses and share the results with the requestor or will construct biomarker covariates and add them to a file with clinical data that is uploaded to an analysis portal so that the requestor can perform the proposed analyses.
