# Supplementary material for: A phase 1b single-arm trial of intratumoral oncolytic virus V937 in combination with pembrolizumab in patients with advanced melanoma: results from the CAPRA study
Source: Cancer Immunol Immunother. 2022 Nov 29;72(6):1405–15. doi: 10.1007/s00262-022-03314-1 (PMC10198910; doi:10.1007/s00262-022-03314-1)
Supplement: Supplementary file 1 — Supplementary file1 (DOCX 92 kb) [file 262_2022_3314_MOESM1_ESM.docx]

**SUPPLEMENTARY MATERIAL**

Protocol

Supplementary Table S1. Multiplex Immunohistochemistry Panel

Supplementary Table S2. Number of Patients Enrolled According to Study Site

Supplementary Figure S1. Patient Disposition

**Supplementary Table S1. Multiplex Immunohistochemistry Panel**

| **Antigen Retrieval** | **Antibody** | **Antibody Dilution** | **Antibody Incubation** | **TSA-Opal (PerkinElmer)** | **TSA-Opal**  **Dilution** | **TSA-Opal**  **Incubation** |
| --- | --- | --- | --- | --- | --- | --- |
| pH9 | FOXP3  (Abcam 236A/E7) | 1:100 | 45 min | 540 | 1:150 | 10 min |
| pH6 | PD-L1  (Cell Signaling E1L3N) | 1:250 | 30 min | 520 | 1:150 | 10 min |
| pH6 | CD8  (Abcam SP16) | 1:50 | 45 min | 570 | 1:150 | 10 min |
| pH6 | CD3  (Abcam SP7) | 1:50 | 45 min | 620 | 1:150 | 10 min |
| pH6 | CD163  (Roche MRQ-26) | Prediluted | 45 min | 650 | 1:150 | 10 min |
| pH6 | Melanoma cocktail  Abcam (HMB45+ M2-7C10 + M2-9E3 + T311) | 1:100 | 45 min | 690 | 1:150 | 10 min |

**Supplementary Table S2. Number of Patients Enrolled According to Study Site**

| **Trial Site** | **No. of Patients Enrolled** |
| --- | --- |
| V937-007-0006: Rutgers Cancer Institute of New Jersey | 31 |
| V937-007-0005: John Wayne Cancer Institute | 4 |
| V937-007-0016: Gabrail Cancer Center Research | 1 |

**Supplementary Figure S1. Patient Disposition**

**
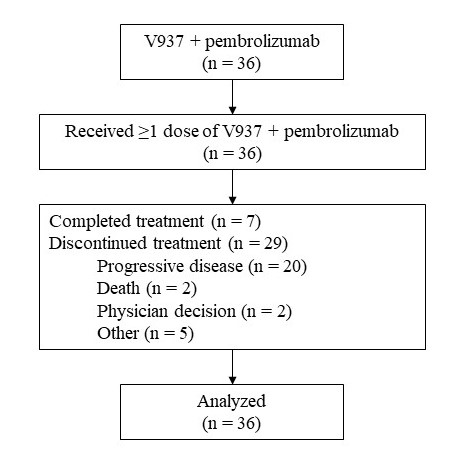
**
