# Supplementary material for: A phase 1b single-arm trial of intratumoral oncolytic virus V937 in combination with pembrolizumab in patients with advanced melanoma: results from the CAPRA study
Source: Cancer Immunol Immunother. 2022 Nov 29;72(6):1405–15. doi: 10.1007/s00262-022-03314-1 (PMC10198910; doi:10.1007/s00262-022-03314-1)
Supplement: Supplementary file 2 — Supplementary file2 (PDF 1551 kb) [file 262_2022_3314_MOESM2_ESM.pdf]

Merck & Co., Inc. policy on posting of redacted study protocols on journal websites is described in Guidelines for Publication of Clinical Trials in the Scientific Literature on the www.merck.com website.

For publicly posted protocols, the Company redacts content that contains proprietary and private information.

This report may include approved and non-approved uses, formulations, or treatment regimens. The results reported may not reflect the overall profile of a product. Before prescribing any product mentioned in this report, healthcare professionals should consult local prescribing information for the product approved in their country.

Copyright © 2022 Merck & Co., Inc., Rahway, NJ, USA and its affiliates. All Rights Reserved. Not for regulatory or commercial use.

CLINICAL PROTOCOL

PHASE I STUDY OF INTRATUMORAL CAVATAK™ (COXSACKIEVIRUS A21) AND PEMBROLIZUMAB IN SUBJECTS WITH ADVANCED MELANOMA

**Protocol Number:**	VLA-011
**Protocol Version:**	3.0
**Protocol Date:**	31 January 2017
**Development Phase:**	1
**Study Sponsor:**	Viralytics Limited<br>Suite 305, Level 3<br>66 Hunter Street<br>Sydney NSW 2000<br>Australia
**FDA IND Number:**	**14547**

CONFIDENTIAL

This document and its contents are the property of and confidential to Viralytics Ltd. Any unauthorized copying or use of this document is prohibited.

SPONSOR APPROVAL SIGNATURE PAGE

Viralytics Ltd has approved of this protocol and assures that that this study will be conducted according to the stipulations described herein.

PPD [redacted]

1/2/2017

Date

Chief Scientific Officer

Viralytics Limited PPD [redacted]

PPD [redacted]

PPD [redacted]

Date

Medical Monitor

PPD [redacted]

February 1, 2017

Date

Project Manager

Viralytics Limited

INVESTIGATOR PROTOCOL AGREEMENT SIGNATURE PAGE

I agree:

To assume responsibility for the proper conduct of the study at this site.

To conduct the study in compliance with this protocol, any future amendments, and with any other study conduct procedures provided by Viralytics Ltd. (Viralytics).

Not to implement any changes to the protocol without written agreement from Viralytics and prior review and written approval from the Research Ethics Committee except where necessary to eliminate an immediate hazard to subjects.

That I am thoroughly familiar with the appropriate use of CVA21 and pembrolizumab, as described in this protocol and any other information provided by Viralytics including, but not limited to, the current CVA21 Investigator's Brochures (IB) and pembrolizumab Package Insert.

That I am aware of, and will comply with, good clinical practices (GCP) and all applicable regulatory requirements.

To ensure that all persons assisting me with the study are adequately informed about CVA21, pembrolizumab and all study-related duties and functions as described in the protocol.

________________________________ __________________

Principal Investigator Signature Date

____

Principal Investigator

TABLE OF CONTENTS

Contents

SPONSOR APPROVAL SIGNATURE PAGE 2
INVESTIGATOR PROTOCOL AGREEMENT SIGNATURE PAGE 3
TABLE OF CONTENTS 4
LIST OF ABBREVIATIONS 9
SYNOPSIS 13
1. Introduction 17
1.1. Background 17
1.1.1 Melanoma 17
1.1.2 Pembrolizumab in Advanced Melanoma 17
1.1.3 CAVATAK™ (Coxsackievirus A21) and Tumor Biology 18
1.2. Rationale for Study 19
1.2.1 Prior Human Experience 19
1.2.2 Other Oncolytic Virus Based Vaccines and Combinations in Melanoma 20
1.2.3 Rationale for Combining CVA21 and Pembrolizumab in Advanced Melanoma 21
2. Study Design 22
2.1. Summary 22
2.2. Study Objectives 23
2.2.1 Primary Objective 23
2.2.2 Secondary Objectives 23
2.2.3 Exploratory Objectives 23
2.3. Criteria for Evaluation 23
2.3.1 Primary Endpoints 23
2.3.2 Secondary Endpoints 23
2.3.3 Exploratory Endpoints 23
2.4. Dose Escalation 23
2.5. Randomization and Blinding 24
3. Study Population 24
3.1. Number of Centers and Participants 24
3.2. Inclusion Criteria 24
3.3. Exclusion Criteria 25

4. Subject Selection and Enrollment 26
4.1. Identifying Participants 26
4.2. Consenting Participants 27
4.2.1 Subject Registration and Numbering 27
4.3. Screening for Eligibility 27
4.4. Ineligible and Non-Recruited Subjects 27
4.5. Withdrawal Procedures 28
4.5.1 Study Treatment Discontinuation 28
4.5.2 Study Discontinuation 28
5. Investigational Medicinal Product 29
5.1. CVA21 29
5.1.1 Dose Preparation 29
5.1.2 Dose Modifications 29
5.1.3 Administration 30
5.1.4 Intra-Subject Dose-Escalation 31
5.2. Pembrolizumab 32
5.2.1 Dose Preparation 32
5.2.2 Dose Modifications 33
5.2.3 Administration 33
5.2.4 Intra-Subject Dose Escalation 33
5.3. Dose-limiting Toxicity 33
5.4. Subject Compliance 33
5.5. Overdose 33
5.6. Prior, Concomitant and Subsequent Therapy 33
5.7. Diet/Pregnancy/Contraception/Other Considerations 34
5.8. Dispensing and Accountability 34
6. Study Assessments 34
6.1. Safety Assessments 34
6.1.1 Physical Examination 35
6.1.2 Vital Signs and Body Weight 35
6.1.3 ECG 35
6.1.4 Brain MRI 35
6.1.5 Laboratory Parameters 35

6.1.6 Pregnancy Test 36
6.1.7 CVA21 Excretion Testing 36
6.1.8 CVA21 Transmission Precautions and Procedures 36
6.2. Efficacy Assessments 38
6.2.1 Imaging 38
6.2.2 Photography 38
6.2.3 QOL/FACT-BRM 38
6.3. Exploratory Assessments 38
6.4. Study Assessments (Visit by Visit Procedures) 39
7. Data Collection 39
7.1. Data Collection and Retrieval 39
7.2. Investigator Reporting Requirements 40
7.3. Record Retention 40
8. Statistics and Data Analysis 40
8.1. Sample Size Calculation 40
8.2. Data Analysis 40
8.2.1 Disposition, Demographics and Baseline Characteristics 40
8.2.2 Efficacy Data 41
8.2.3 Safety Data 41
9. Adverse Events 42
9.1. Definitions 42
9.2. Reporting Overdoses 43
9.3. Reporting of Pregnancy 44
9.4. Recording AEs and SAEs 44
9.5. Evaluation of AEs an dSAEs 44
9.5.1 Assessment of Seriousness 44
9.5.2 Assessment of Causality 44
9.5.3 Assessment of Severity 45
9.5.4 Assessment of Expectedness 46
9.6. Reporting of SAE/SARs and SUSARs 46
9.7. Regulatory Reporting Requirements 46
9.7.1 Annual Report 46
9.7.2 Expedited Reporting 47

9.8. Follow-up Procedures 47
10. Trial Management and Oversight 47
10.1. Data Monitoring Committee 47
10.2. Inspection of Records/Source Data 48
10.3. Monitoring 48
10.4. Risk Management 48
10.4.1 Potential Risks 48
10.4.2 Minimizing Risks 49
11. Good Clinical Practice 49
11.1. Ethical Conduct of the Study 49
11.2. Regulatory Compliance 50
11.3. Investigator Responsibilities 50
11.3.1 Informed Consent 50
11.3.2 Study Site Staff 50
11.3.3 Data Recording 50
11.3.4 Investigator Documentation 51
11.3.5 GCP Training 51
11.3.6 Confidentiality 51
11.3.7 Data Protection 51
12. Study Conduct Responsibilities 51
12.1. Protocol Amendments 51
12.2. Protocol Violations and Deviations 52
12.3. Study Record Retention 52
12.4. End of Study/Study Termination by Sponsor 52
12.5. Audits and Inspections 52
12.6. Continuation of Drug Following End of Study 53
13. Reporting, Publications and Notification of Results 53
13.1. Authorship Policy 53
13.2. Publication 53
14. References 54
15. Appendices 56
15.1. Schedule of Procedures 56
15.2. ECOG Performance Status 59

15.3. KEYTRUDA™ (Pembrolizumab) Package Insert 60
15.4. CVA21 Intratumoral Injection Technique 61
15.5. Listing of Auto-Immune Diseases 67
15.6. Evaluation of Response 69
15.7. Monitoring Plan, Quality Control and Quality Assurance 71
15.8. Healthcare Worker Instructions in case of Suspected CVA21 Transmission 72
15.9. Record of Illness by Health Care Worker 74
15.10. Instructions for Close Contacts for CVA21 Transmission 75
15.11. Record of Illness by Close Contact 76
15.12. FACT-BRM Questionnaire 77

LIST OF ABBREVIATIONS

IV	Roman numeral 4, as in stage IV disease.
ACTH	Adrenocorticotropic hormone
ADL	Activities of Daily Living
AE	Adverse Event
ALT	Alanine Aminotransferace
AR	Adverse Reaction
AST	Aspartate Aminotransferace
ANC	Absolute neutrophil count
BRAF	A human gene that encodes a protein called B-Raf
BSL	Bio-Safety Level
CFR	Code of Federal Regulations (FDA)
cGMP	Current Good Manufacturing Practices
CNS	Central Nervous System
CRO	Contract Research Organization
CT	Computed tomography
CTCAE	Common Terminology Criteria for Adverse Events
CTLA-4	Cytotoxic T-Lymphocyte-Associate Protein 4
CVA21	Coxsackievirus A21
DAF	Decay-accelerating factor
DLT	Dose Limiting Toxicity
DMC	Data Monitoring Committee
DOR	Duration of Response
DSUR	Development Safety Update Report
DTAP	Diethylenetriaminepentaacetic Acid
ECG	Electrocardiogram

ECOG	Eastern Cooperative Oncology Group
eCRF	Electronic case Report Form
EDC	Electronic Data Capture
FACT-BRM	Functional Assessment of Cancer – Biological Response Modifier (QOL)
FDA	Food and Drug Administration
GCP	Good Clinical Practice
GM-CSF	Granulocyte Macrophage Colony-Stimulating Factor
Hgb	Hemoglobin
HIV	Human Immunodeficiency Virus
HLA	Human leucocyte antigen
HLP	Hind Limb Paralysis
IBC	Institutional Biosafety Committee (or equivalent)
ICAM-1	Intercellular adhesion molecule-1
IND	Investigational New Drug application
INR	International normalized ratio
IRB	Institutional Review Board
irCR	Immune-related Complete Response
irPD	Immune-related Progressive Disease
irPR	Immune-related Partial Response
irRC	Immune-related Response Criteria
irSD	Immune-related Stable Disease
ISF	Investigator Site File
IT	Inratumoral
IUD	Intrauterine Device
IV	Intravenous (sometimes also Roman numeral 4, as in stage IV disease)

LDH	Lactate dehydrogenase
LH	Luteinizing Hormone
LN	Lymph Node
MedDRA	Medical Dictionary for Regulatory Activities
MRI	Magnetic resonance imaging
NOEL	No-Observed Adverse-Effect Level
ORR	Objective Response Rate
OTC	Over the Counter
PBMC	Peripheral blood mononuclear cells
PBS	Phosphate-buffered saline
PD-1	Programmed death receptor-1
PD-L1	Programmed Death Ligand-1
PFS	Progression Free Survival
PET	Positron emission tomography
PT	Prothrombin time
PTT	Partial thromboplastin time
QA	Quality Assurance
QOL	Quality of life
RT	Room temperature
REC	Research Ethics Committee
RECIST	Response Criteria in Solid Tumors
RNA	Ribonucleic Acid
SAE	Serious Adverse Event
SAD	Short Axis Diameter (used in measuring Lymph Nodes)
SAP	Statistical Analysis Plan
SAR	Serious Adverse Reaction

SC	subcutaneous
SOP	Standard Operating Procedures
SPD	Sum of the perpendicular diameters (Used in measuring index lesions)
SUSAR	Suspected Unexpected Serious Adverse Reaction
$TCID_{50}$	50% tissue culture infectious dose
TEAE	Treatment Emergent Adverse Event
TMF	Trial Master File
TSH	Thyroid stimulating hormone
ULN	Upper limit of normal
WBC	White Blood Count
WHO	World Health Organization
WNL	Within normal limits

SYNOPSIS

**Study Title**	PHASE I STUDY OF INTRATUMORAL CAVATAK™ (COXSACKIEVIRUS A21) AND PEMBROLIZUMAB IN SUBJECTS WITH ADVANCED MELANOMA
**Protocol Number**	VLA-011
**Study Sites**	Approximately 4 to 6 sites in the USA
**Planned Sample Size**	Up to 50 subjects
**Study Phase**	Phase 1b
**Objectives**	**Primary Objectives:**<br>To assess the safety and tolerability of intravenous pembrolizumab in combination with intratumoral CVA21 by incidence of dose-limiting toxicities (DLT).<br>**Secondary Objectives:**<br>1. To assess the clinical efficacy of pembrolizumab in combination with intratumoral CVA21 in terms of immune-related progression-free survival (irPFS) at 12 months, PFS hazard ratio, overall response rate (ORR), 1-year survival, overall survival (OS) and quality of life.<br>2. Assess the response of injected and non-injected melanoma deposits after CVA21 and pembrolizumab.<br>3. Assess the time to initial response.<br>4. Assess the durable response rate.<br>CCI [redacted]
**Study Design**	Open label, single arm study of intratumoral CVA21 and pembrolizumab in advanced melanoma patients.
**Criteria for Evaluation**	**Primary Endpoints:**<br>The primary study endpoints are the occurrence of AEs, SAEs, and dose limiting toxicities (DLTs).<br>**Secondary Endpoints:**<br>The main secondary efficacy endpoint is best response as assessed by WHO criteria modified for immune-related response. Other secondary efficacy endpoints are irPFS, OS, time to response, duration of response, and change in the quality of life assessments.<br>CCI [redacted]

**Eligibility Criteria**	**Inclusion Criteria:**<br>1. Subjects with metastatic or unresectable stage IIIb/c or IV melanoma for whom treatment with pembrolizumab is indicated and who have at least one cutaneous, subcutaneous tumor or palpable lymph node amenable to intratumoral injection.<br>2. Subjects must have histological confirmation of melanoma, which will be required by previous biopsy or cytology.<br>3. At least one tumor must qualify to be an index lesion for modified WHO criteria.<br>4. No chemotherapy, radiation therapy, hormonal treatment or immunotherapy within 28 days prior to initiation of treatment. Subjects must have resolution of toxic effects of the most recent chemotherapy to grade 1 or less (except alopecia). If the subject received major surgery or radiation therapy of > 30Gy, they must have recovered from the toxicity and/or any complications.<br>5. Subjects must have adequate hematologic, hepatic and renal function, defined as:<br>a. Absolute neutrophil count (ANC) > 1.5 x 10^9/L<br>b. Platelets ≥ 100 x 10^9/L,<br>c. PT ≤ 1.5 x the upper limit of normal (ULN)<br>d. PTT ≤ 1.5 x ULN<br>e. INR ≤ 1.5 x ULN<br>f. Total bilirubin ≤ 1.5 times the upper limit of normal (ULN)<br>g. Aspartate aminotransferase (AST) and alanine aminotransferase (ALT) ≤ 2.5 x ULN or ≤ 5 x ULN for patients with liver mets<br>h. LDH ≤ 2.5 x ULN<br>i. Serum creatinine ≤ 1.5xULN <u>OR</u> ≥45 mL/min/1.73m^2 by **CKD-EPI equation** for subjects with creatinine levels >1.5x institutional ULN OR GFR ≥ 45mL/min/1.73m2 by CKD-EPI equation or MDRD equation.<br>6. Male or female age 18 years or older.<br>7. Performance status (Eastern Cooperative Oncology Group [ECOG]) 0 or 1<br>8. Subjects must be willing to provide written, informed consent.<br>9. No active bleeding.<br>10. Anticipated lifespan greater than 12 weeks<br><br>**Exclusion Criteria:**<br>1. Ocular primary tumors.<br>2. Presence of any central nervous system (CNS) tumor that has not been stable for at least 4 weeks off corticosteroids and confirmed by imaging.<br>3. Subjects with tumors close to an airway, major blood vessel or spinal cord that, in the opinion of the Investigators, could cause occlusion or compression in the case of tumor swelling or erosion into a major vessel in the case of necrosis. Subjects with lesions in mucosal areas (vulvar, anal, oral cavity, etc.) are eligible, as long as the subject has at least one lesion suitable for injection. Consult the Viralytics Medical Monitor for confirmation.<br>4. Subjects with active, known or suspected autoimmune or immunosuppressive disease (see Appendix 15.5). Hypothyroid patients on adequate thyroid replacement who are asymptomatic and have normal thyroid function tests are eligible.<br>5. Subjects previously treated with Coxsackievirus A21.

6. Subjects with a condition requiring systemic treatment with either corticosteroids or other immunosuppressive medications within 14 days prior to first treatment. Low dose replacement steroids administered prior to imaging may be considered. Consult the Viralytics Medical Monitor for confirmation.
7. Clinically significant (that is, active) cardiovascular disease: cerebral vascular accident/stroke (< 6 months prior to enrollment), myocardial infarction (< 6 months prior to enrollment), unstable angina, congestive heart failure (New York Heart Association Classification Class ≥ II), or serious cardiac arrhythmia requiring medication.
8. Female participants of childbearing potential must have a negative urine or serum pregnancy test and confirmed within 10 days before starting study treatment and must be willing to use two adequate barrier methods of contraception starting with the screening visit through 120 days after the last dose of pembrolizumab.
9. Male participants with a female partner(s) of child-bearing potential must be willing to use two adequate barrier methods of contraception from screening through 120 days after the last dose of pembrolizumab.
10. Subject requiring or other investigational agents while on treatment in this trial.
11. History of other malignancy within the past 3 years with the following exceptions:
    a. malignancy treated with curative intent and with no known active disease present for > 3 years before enrollment and felt to be at low risk for recurrence by the treating physician and approved by the Viralytics Medical Monitor.
    b. adequately treated non-melanoma skin cancer or lentigo maligna without evidence of disease.
    c. adequately treated cervical carcinoma in situ without evidence of disease.
    d. adequately treated breast ductal carcinoma in situ without evidence of disease.
    e. prostatic intraepithelial neoplasia without evidence of prostate cancer.
    f. adequately treated urothelial papillary non-invasive carcinoma or carcinoma in situ.
    g. Adequately treated secondary malignancy such as chronic leukemias or indolent lymphomas without evidence of current disease may be considered. Consult the Viralytics Medical Monitor for confirmation.
12. Active infection requiring systemic therapy.
13. Known history of human immunodeficiency virus (HIV) disease, active hepatitis B or hepatitis C.
14. Subject has known sensitivity to any of the products or components to be administered during dosing.
15. Subject likely to not be available or capable of completing all protocol-required visits or procedures.
16. History or evidence of other clinically significant disorders, condition or disease that, in the opinion of the investigator or Viralytics Medical Monitor, would pose a risk to subject safety or interfere with the planned protocol evaluation.
17. Inability to give informed consent and comply with the protocol. Subjects with a history of psychiatric illness must be judged able to understand

	fully the investigational nature of the study and the risks associated with the therapy.<br>18. Any medical condition that in the opinion of the Principal Investigator would compromise safety or conduct of the study procedures.
**Investigational Therapy**	CAVATAK™ (Coxsackievirus A21 or CVA21)
**Reference Therapy**	Historical pembrolizumab alone data
**Treatment Duration**	Subjects will receive a maximum of 19 sets of CVA21 injections and pembrolizumab administered every 3 weeks starting on Day 8 and continuing every 3 weeks, up to 2 years.
**Statistical Methods and Planned Analyses**	Simon's optimal two-staged design will be applied with the objective of estimating overall response rate. Safety of CVA21 will be assessed and the study stopped early for subject safety according to stopping rules.

1. Introduction

1.1. Background

1.1.1 Melanoma

The American Cancer Society estimates that there will be over 76,000 new diagnoses and 9,700 deaths from melanoma in the United States in 2014.[1] Metastatic melanoma has a poor prognosis with less than 5% of subjects surviving five years from the manifestation of visceral organ involvement. Disease-specific survival curves in all stages of melanoma have a negative slope, indicative that metastatic disease can develop many years after the initial diagnosis even from thin primary lesions. For instance, in survival data compiled by Balch et al, up to 10% of patients presenting with stage I melanoma (primary site less than 1 mm in depth and no nodal involvement) will die as a consequence of metastatic disease within 10 years.[2] Disease recurrence can manifest years or even decades after the initial diagnosis. The potential lethality of early stage melanoma distinguishes it from other solid tumors.

Immunotherapy has been studied for decades to treat melanoma and the only FDA-approved immunotherapeutic agent for metastatic melanoma was interleukin-2 until 2011. There have been recent significant advances in the treatment of melanoma. Two randomized phase III studies have shown improved survival for s u b j e c t s with advanced melanoma treated with ipilimumab.[3, 4] The FDA approved the use of ipilimumab for first or second-line treatment of metastatic melanoma in March 2011. Combined PD-1/PD-L1 blockade has significant activity and combinations of T-cell checkpoint inhibitors are also showing significant clinical promise. The combination of nivolumab (anti-PD-1) and ipilimumab showed an objective response rate of 40% in subjects with metastatic melanoma.[5, 10] More recently (September 2014), the FDA approved the use of pembrolizumab (anti-PD-1) for advanced melanoma patients who have failed either B-Raf targeted therapy or ipilimumab treatment. In December 2014, the FDA also approved nivolumab, another anti-PD-1 targeted monoclonal antibody. Targeted therapy in melanoma has also shown promise. Vemurafenib, which targets the BRAF V600E mutation, has an objective response rate of approximately 50%.[6, 9] A phase III study comparing vemurafenib to dacarbazine showed a significant increase in survival for subjects receiving vemurafenib. The median progression-free survival was 5.3 months in the vemurafenib group, leading to FDA approval in August 2011.[7] Similar findings have been observed with another BRAF inhibitor, dabrafenib, used alone or in conjunction with trametinib. Both agents have recently garnered FDA approval as single agents or in combination.[8]

Even with the recent FDA accelerated approval of pembrolizumab displaying an ORR of 24% and nivolumab an ORR of 32% in subjects progressing following B-Raf targeted therapy or ipilimumab, there is still a substantial unmet need for new successful therapies in patients with advanced melanoma.

1.1.2 Pembrolizumab in Advanced Melanoma

Pembrolizumab is a human programmed death receptor-1 (PD-1)-blocking antibody indicated for the treatment of patients with unresectable or metastatic melanoma and disease progression following ipilimumab and, if BRAF V600 mutation positive, a BRAF inhibitor. Pembrolizumab is a highly selective, humanized monoclonal IgG4–kappa isotype antibody targeting PD-1 that

functions to block the negative immune regulatory signaling of the PD-1 receptor expressed by T cells.[11-13]

FDA accelerated approval was based on the results of a multicenter, open-label, randomized (1:1), dose comparative, activity-estimating cohort conducted within the Keynote-001 trial. In this treatment cohort, 173 subjects with unresectable or metastatic melanoma with disease progression within 24 weeks of the last dose of ipilimumab and, if BRAF V600 mutation positive, prior treatment with a BRAF inhibitor, were randomized to receive pembrolizumab at either 2 mg/kg (n=89) or 10 mg/kg (n=84) intravenously once every 3 weeks until disease progression or unacceptable toxicity.

The Keynote-001 endpoints were confirmed overall response rate (ORR) according to Response Evaluation Criteria in Solid Tumors (RECIST v1.1) and duration of response (DOR). The ORR was 24% (95% CI: 15, 34) in the 2 mg/kg arm, consisting of one complete response and 20 partial responses.[10] Objective responses were observed in 21 subjects, 3 (14%) had disease progression at 2.8, 2.9, and 8.2 months after initial response. The remaining 18 subjects (86%) have ongoing responses, ranging from 1.4+ to 8.5+ months; 8 subjects have ongoing responses of 6 months or longer. Comparable ORR results were observed in the 10 mg/kg arm.

The most common (≥ 20%) treatment related adverse reactions among subjects receiving pembrolizumab 2 mg/kg every 3 weeks were fatigue, cough, nausea, pruritus, rash, decreased appetite, constipation, arthralgia, and diarrhea. Appendix 15.3 contains information for management of pembrolizumab.

1.1.3 CAVATAK™ (Coxsackievirus A21) and Tumor Biology

Coxsackievirus A21 (CVA21, CAVATAK) is a naturally occurring virus that induces mild upper respiratory symptoms during natural infection of humans. CVA21 naturally infects cells in the respiratory tract, which are known to express ICAM-1, thereby resulting in the development of "common cold"-like symptoms frequently observed during natural and experimental infection.[14-17] CVA21 also displays potent oncolytic activity against both *in vitro* cultures of human cancer cells and against *in vivo* xenografts of human cancers in mouse models (melanoma,[18] prostate cancer,[19] breast cancer,[20] and multiple myeloma).[21] In the mouse xenograft CVA21 challenge models, progeny virus released from infected cells is capable of infecting adjacent cells, entering the systemic circulation, and targeting micrometastatic foci. Furthermore, tumor antigens released following CVA21-induced cancer cell lysis may potentially stimulate the host immune system against such neoplastic cells.

CVA21 is a live bio-selected oncolytic virus preparation derived from the non-genetically altered prototype *Kuykendall* strain of Coxsackievirus A21 propagated in cell cultures. Coxsackieviruses are non-enveloped viruses with positive single-stranded RNA and 4 capsid proteins. The CVA21 capsid contains a single- stranded RNA positive sense genome of 7405 nucleotides, encoding an open reading frame of 2208 codons. Also within the viral genome is a 713 nucleotide 5' non-coding region and 3' non-coding region of 68 nucleotides attached to a poly (A) tract. The CVA21 capsid is approximately 28 nm in diameter, allowing the purified virus to pass through a sterilizing (bacterial and fungal) 0.2-µm filter for aseptic processing and dispensing of the finished product.

1.2. Rationale for Study

A series of preclinical studies have been completed. Animal data confirm that CVA21 can cause oncolysis of tumors at sites distant to the primary site of viral administration (abscopal effect). Intratumoral and IV administration of CVA21 into mice bearing ICAM- 1 and/or DAF-expressing xenografts showed significant decreases in the injected primary tumor volumes of virus-treated mice compared to PBS-treated control mice.

Based on the results from the repeat-dose toxicology study on CVA21 administered subcutaneously (SC) to male and female chimeric Hu/Mu ICAM-1 transgenic Balb/C mice, the administered SC dose of about 1.25 x 10^9 TCID50/kg caused no apparent toxic effects, including any signs of hind-limb paralysis (HLP) or other forms of myositis, and was considered to be the no-observed adverse-effect level (NOAEL) dose for the study. This CVA21 SC dose in mice has a human equivalent dose of about 1 x 10^8 $TCID_{50}$/kg, which provides a safety margin of about 20-fold for the human CVA21 dose of about 4.5 x 10^6 $TCID_{50}$/kg (for a 70-kg patient) to be evaluated during the present clinical trial. Furthermore, Hu/Mu ICAM-1 transgenic mice tolerated systemic exposure to CVA21 at levels of approximately 8 x 10^4 TCID50/mL equivalents post-primary SC injection (30 minutes after dosing), which is approximately 50 times the level (1.8 x 10 to 1.2 x 10^3 $TCID_{50}$/mL equivalents) observed in the serum of subjects in earlier clinical trials following the initial intratumoral (IT) injection with CVA21 at 3.2 x 10^8 $TCID_{50}$.

1.2.1 Prior Human Experience

To date there are 3 completed phase I clinical studies assessing the intratumoral administration of CVA21 in 14 Subjects with stage IV melanoma. With respect to previous CVA21 clinical experience, 14 subjects with stage IV malignant melanoma have been administered either 1 or 2 intratumoral injections of CVA21 in Australian hospitals within 3 separate phase I studies.

The first clinical use of CVA21 as an oncolytic agent was initiated by the Sponsor late in 2003 with the intratumoral administration of CVA21 to two subjects with late-stage melanoma (PSX-X01). Subsequently, an additional small single-dose study was performed to formally assess the safety of intratumoral

CVA21 administration and to gather further preliminary information about viral persistence and anti-CVA21 antibody production (PSX-X02). A Phase 1 dose escalation study was undertaken to assess the safety of multiple intratumoral administrations of increasing concentrations of CVA21 in subjects with late-stage melanoma. Secondary objectives of that study also addressed the response of tumor size, systemic exposure, viral persistence, and anti-CVA21 antibody production (PSX-X03). In addition, the Sponsor conducted 2 phase 1 studies involving administration of CVA21 to subjects with late-stage cancer. In the first study, the intravenous administration of CVA21 was investigated in 10 subjects with late-stage melanoma, breast, colon, or prostate cancer (PSX-X04). In the second study the intratumoral administration of CVA21 was assessed in 4 subjects with recurrent squamous cell carcinoma of the head and neck (VLA-X06). In these phase I studies, CVA21 administration by either IV or IT routes was generally well tolerated, with evidence of disease stabilization in some subjects.

Seventy (70) subjects were enrolled in an open-label multi-center phase II study of the IT administration of CVA21 to subjects with stage IIIC and stage IV melanoma (VLA-007:

ClinicalTrials.gov Identifier: NCT01227551). CVA21 was administered on days 1, 3, 5, 8, 22, 43, 64, 85, 106, and 127. Responding subjects were eligible to receive maintenance administrations (VLA-008: ClinicalTrials.gov Identifier: NCT01636882). A total of 70 subjects have received IT administration. Follow-up and treatments are ongoing. Subjects have (57 from the original study and 13 from a biopsy sub study) received an average of 8.3 injections, with the most common side effects being grade 1 fatigue, chills, local injection site reactions, and fever. There have been no reports of grade 3 or 4 toxicities related to CVA21. The final results of this study were presented in abstract form indicating that the primary endpoint of immune-related progression-free survival at 6 months was greater than 22.5% had been achieved (38.6%). Furthermore, the study displayed an overall tumor response of 28.1% using immune-related response evaluation in solid tumor (RECIST 1.1) criteria. Abscopal responses in cutaneous, subcutaneous, lymph node and lung lesions have been observed in a number of subjects. The mechanism of response is currently being investigated, but likely involves a combination of viral-mediated oncolysis and host-immune cell activation involving interferon-γ production by T cells. Biopsy studies are ongoing to investigate injected and non-injected melanoma tumors after CVA21 administration to ascertain if phenotypic or functional changes occur in tumor infiltrating T cells and whether the upregulation of checkpoint inhibitor molecules such as PD-1 may influence response.

1.2.2 Other Oncolytic Virus Based Vaccines and Combinations in Melanoma

There have been many studies of oncolytic viruses in melanoma. Some examples include herpes simplex virus, poxvirus, vaccinia, and reovirus.[22-25] The results of a phase III trial with a genetically modified herpes simplex virus (HSV) known as Talimogene laherparepvec (T-VEC) were reported at the 2014 American Society of Clinical Oncology Annual Meeting. The genetic modifications incorporated into T-VEC include the ability of the HSV to secrete GM- CSF. The genes encoding ICP34.5 and ICP47 were deleted, which enhance the tumor lytic properties of this virus. The trial had a 2:1 randomization to T-VEC versus GM-CSF; 436 subjects were in the intent-to-treat group with 295 assigned to receive T-VEC and 141 GMCSF.[26] Table 1 summarizes the results of this trial.

Table 1: Results of the Phase 3 T-VEC versus GM-CSF study

	**GM-CSF**	**T-VEC**
Objective Response	5.7 %	26.4 %
CR	0.7 %	10.8 %
PR	5.0 %	15.6 %
Durable Response	2.1 months	16.3 months (p< 0.0001)
Overall Survival	18.9 months	23.3 months (p = 0.51)

A phase IB study was also presented studying the combination of T-VEC with anti-CTLA-4 ipilimumab.[27] The rationale for combining an oncolytic vaccine with a T-cell checkpoint inhibitor

was the hypothesis that existing T cell anti-tumor responses are expanded by ipilimumab.[28] T-VEC given before ipilimumab would expand the T-cell repertoire for melanoma antigens and subsequent ipilimumab could increase the lifespan and activity of these melanoma-specific T cells. T-VEC was given on weeks 1 and 4, then every 2 weeks thereafter. Ipilimumab (3 mg/kg IV) started at week 6 (after 3 T-VEC doses) and continued every 3 weeks for a total of 4 doses, which is the standard dose and schedule for this agent. Nineteen subjects enrolled and 10 had stage IV (M1b/c disease). All subjects had at least one tumor deposit amenable to intratumoral injection. Toxicities were as anticipated with 32% of subjects experiencing grade 3 or 4 toxicities attributable to ipilimumab. The objective response was 57% with 6 subjects achieving complete response, 5 with partial response, and 6 with disease stability. The median time to response was 2.9 months, which is shorter than anticipated with ipilimumab or T-VEC alone. There was significant expansion in the peripheral blood of CD8+ T cells of 1.8-fold after T-VEC and 2.9-fold after ipilimumab was completed. A larger randomized trial is currently in progress to confirm these preliminary findings.

Although the combination study was small, these results help to establish the proof of concept that oncolytic viruses injected into subcutaneous melanoma deposits can initiate a cellular immune response that can be amplified by immune checkpoint inhibition, resulting in clinically meaningful responses and a much higher objective response than anticipated with either agent used as monotherapy. In addition to ipilimumab, a Phase I/II study combining intra-lesional T-VEC and intravenous pembrolizumab in advanced melanoma subjects (n=110) has commenced patient treatment.

1.2.3 Rationale for Combining CVA21 and Pembrolizumab in Advanced Melanoma

As summarized above, there is a strong rationale for combining oncolytic virus vaccination with immune checkpoint inhibition and initial clinical experience with this approach is promising. This result also supports the growing consensus that the effectiveness of extant T-cell enhancing immunotherapies such as pembrolizumab, ipilimumab and interleukin-2 are dependent on pre-existing T-cell recognition of melanoma. In recent preclinical studies performed in a fully immune-competent mouse model of melanoma, enhanced anti-tumor activity was observed in mice receiving a combination of intratumoral CVA21 and immune-checkpoint antibody (anti-PD-1) compared to activity displayed from single agent use alone (Figure 1). [29]

Figure 1: Immune competent mouse model of melanoma of CVA21 in combination with anti-PD-1, spider plot of individual tumor growth

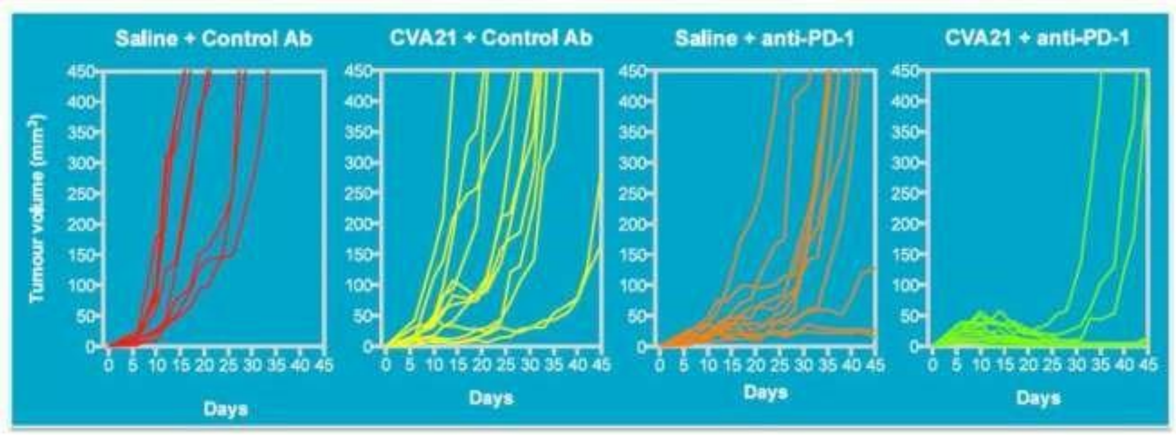

The present study will employ a phase Ib design using the established dose of CVA21 with pembrolizumab in subjects with advanced melanoma for whom pembrolizumab would be considered standard of care. Our hypothesis is that oncolysis of melanoma cells by CVA21 will be important in amplifying the T-cell potentiating effects of pembrolizumab. Subject treatment will consist of intratumoral CVA21 administrations on trial days 1, 3, 5, 8 and at 3-weekly intervals (up to a maximum of 19 total injections) with intravenous pembrolizumab (2 mg/kg solution) starting on day 8 and continuing every 3 weeks, up to 2 years depending on response until complete response, disease progression per modified WHO criteria, or intolerance of study treatment, whichever occurs first. The maximum dose of CVA21 for this study is 3 x 10^8 $TCID_{50}$ (about 4.5 x 10^6 $TCID_{50}$/kg for a 70-kg subject) by intratumoral administration.

In addition to monitoring for toxicity and clinical response, blood samples and tumor samples (optional) will be obtained to assess immunologic measures relevant to melanoma immune responses and pembrolizumab T-cell checkpoint inhibition (see Section 10.4 for more details about immune monitoring).

2. Study Design

2.1. Summary

This is an open-label study intended to enroll a maximum of 50 subjects. Subjects will start treatment with intratumoral CVA21 administered on trial Days 1, 3, 5 and 8 followed by administration of both CVA21 and intravenous pembrolizumab (2 mg/kg solution) starting on Day 8 and continuing every 3 weeks, up to 2 years depending on response until complete response, disease progression per modified WHO criteria, or intolerance of study treatment, whichever occurs first. On days where both CVA21 and pembrolizumab are administered, CVA21 will be administered first. The maximum dose of CVA21 for this study is 3 x 10^8 $TCID_{50}$ (about 4.5 x 10^6 $TCID_{50}$/kg for a 70-kg subject) by intratumoral (IT) administration. Additional CVA21 injections will be administered on Days 106 and every 3 weeks thereafter to a maximum of 19 injections.

Tumors lying in mucosal regions or close to an airway, major blood vessel, or spinal cord that, in the opinion of the Investigators, could cause occlusion or compression in the case of tumor swelling or erosion into a major vessel in the case of necrosis are NOT to be injected with CVA21.

If the Investigator believes that an excision of a lesion is in the best interest of a study subject, the site should contact the Viralytics Medical Monitor for further discussion prior to performing the procedure so the impact on efficacy analysis may be assessed.

2.2. Study Objectives

2.2.1 Primary Objective

To assess the safety and tolerability of intravenous pembrolizumab in combination with intratumoral CVA21 by incidence of dose-limiting toxicities (DLT).

2.2.2 Secondary Objectives

1. To assess the clinical efficacy of pembrolizumab in combination with intratumoral CVA21 in terms of immune-related progression-free survival (irPFS) at 12 months, PFS hazard ratio, overall response rate (ORR), 1-year survival, overall survival (OS) and quality of life.
2. Assess the response of injected and non-injected melanoma deposits after CVA21 and pembrolizumab.
3. Assess the time to initial response.
4. Assess the durable response rate.

2.2.3 Exploratory Objectives

These assessments will only be conducted at Rutgers Cancer Institute:

1. Assess peripheral blood for changes in T-cell phenotypes after CVA21 and pembrolizumab.
2. Assess for T-cell immune response to known melanoma antigens during treatment.

2.3. Criteria for Evaluation

2.3.1 Primary Endpoints

The primary study endpoints are the occurrence of AEs, SAEs, and dose limiting toxicities (DLTs).

2.3.2 Secondary Endpoints

The main secondary efficacy endpoint is best response as assessed by WHO criteria modified for immune-related response. Other secondary efficacy endpoints are irPFS, OS, time to response, duration of response, and change in the quality of life assessments.

2.3.3 Exploratory Endpoints

The exploratory endpoints of interest are the immune monitoring measures of T cell and other cell counts from blood and tissue samples.

2.4. Dose Escalation

Dose escalations will not be permitted.

2.5. Randomization and Blinding

Not applicable; this is an open-label, non-randomized study.

3. Study Population

3.1. Number of Centers and Participants

This study will involve approximately 4 to 6 clinical study sites selected in the United States and will enroll a maximum of 50 subjects.

3.2. Inclusion Criteria

1. Subjects with metastatic or unresectable stage IIIb/c or IV melanoma for whom treatment with pembrolizumab is indicated and who have at least one cutaneous, subcutaneous tumor or palpable lymph node amenable to intratumoral injection.
2. Subjects must have histological confirmation of melanoma, which will be required by previous biopsy or cytology.
3. At least one tumor must qualify to be an index lesion for modified WHO criteria.
4. No chemotherapy, radiation therapy, hormonal treatment or immunotherapy within 28 days prior to initiation of treatment. Subjects must have resolution of toxic effects of the most recent chemotherapy to grade 1 or less (except alopecia). If the subject received major surgery or radiation therapy of > 30Gy, they must have recovered from the toxicity and/or any complications.
5. Subjects must have adequate hematologic, hepatic and renal function, defined as:
    a. Absolute neutrophil count (ANC) > 1.5 x 10^9/L
    b. Platelets ≥ 100 x 10^9/L,
    c. PT ≤ 1.5 x the upper limit of normal (ULN)
    d. PTT ≤ 1.5 x ULN
    e. INR ≤ 1.5 x ULN
    f. Total bilirubin ≤ 1.5 times the upper limit of normal (ULN)
    g. Aspartate aminotransferase (AST) and alanine aminotransferase (ALT) ≤ 2.5 x ULN or ≤ 5 x ULN for patients with liver metastases
    h. LDH ≤ 2.5 x ULN
    i. Serum creatinine ≤ 1.5xULN <u>OR</u> ≥45 mL/min/1.73m^2 by **CKD-EPI equation** for subjects with creatinine levels >1.5x institutional ULN OR GFR ≥ 45mL/min/1.73m2 by CKD-EPI equation or MDRD equation.
6. Male or female age 18 years or older.
7. Performance status (Eastern Cooperative Oncology Group [ECOG]) 0 or 1 (Appendix 15.2).
8. Subjects must be willing to provide written, informed consent.

9. No active bleeding.
10. Anticipated lifespan greater than 12 weeks.

3.3. Exclusion Criteria

1. Ocular primary tumors.
2. Presence of any central nervous system (CNS) tumor that has not been stable for at least 4 weeks off corticosteroids and confirmed by imaging.
3. Subjects with tumors close to an airway, major blood vessel or spinal cord that, in the opinion of the Investigators, could cause occlusion or compression in the case of tumor swelling or erosion into a major vessel in the case of necrosis. Subjects with lesions in mucosal areas (vulvar, anal, oral cavity, etc.) are eligible, as long as the subject has at least one lesion suitable for injection. Consult the Viralytics Medical Monitor for confirmation.
4. Subjects with active, known or suspected autoimmune or immunosuppressive disease (see Appendix 15.5). Hypothyroid patients on adequate thyroid replacement who are asymptomatic and have normal thyroid function tests are eligible.
5. Subjects previously treated with Coxsackievirus A21.
6. Subjects with a condition requiring systemic treatment with either corticosteroids or other immunosuppressive medications within 14 days prior to first treatment. Low dose replacement steroids administered prior to imaging may be considered. Consult the Viralytics Medical Monitor for confirmation.
7. Clinically significant (that is, active) cardiovascular disease: cerebral vascular accident/stroke (< 6 months prior to enrollment), myocardial infarction (< 6 months prior to enrollment), unstable angina, congestive heart failure (New York Heart Association Classification Class ≥ II), or serious cardiac arrhythmia requiring medication.
8. Female participants of childbearing potential must have a negative urine or serum pregnancy test and confirmed within 10 days before starting study treatment and must be willing to use two adequate barrier methods of contraception starting with the screening visit through 120 days after the last dose of pembrolizumab.
9. Male participants with a female partner(s) of child-bearing potential must be willing to use two adequate barrier methods of contraception from screening through 120 days after the last dose of pembrolizumab.
10. Subject requiring or other investigational agents while on treatment in this trial.
11. History of other malignancy within the past 3 years with the following exceptions:
    a. malignancy treated with curative intent and with no known active disease present for > 3 years before enrollment and felt to be at low risk for recurrence by the treating physician and approved by the Viralytics Medical Monitor.
    b. adequately treated non-melanoma skin cancer or lentigo maligna without evidence of disease.

c. adequately treated cervical carcinoma in situ without evidence of disease.

d. adequately treated breast ductal carcinoma in situ without evidence of disease.

e. prostatic intraepithelial neoplasia without evidence of prostate cancer.

f. adequately treated urothelial papillary non-invasive carcinoma or carcinoma in situ.

g. Adequately treated secondary malignancy such as chronic leukemias or indolent lymphomas without evidence of current disease may be considered. Consult the Viralytics Medical Monitor for confirmation.

12. Active infection requiring systemic therapy.
13. Known history of human immunodeficiency virus (HIV) disease, active hepatitis B or hepatitis C.
14. Subject has known sensitivity to any of the products or components to be administered during dosing.
15. Subject likely to not be available or capable of completing all protocol-required visits or procedures.
16. History or evidence of other clinically significant disorders, condition or disease that, in the opinion of the investigator or Viralytics Medical Monitor, would pose a risk to subject safety or interfere with the planned protocol evaluation.
17. Inability to give informed consent and comply with the protocol. Subjects with a history of psychiatric illness must be judged able to understand fully the investigational nature of the study and the risks associated with the therapy.
18. Any medical condition that in the opinion of the Principal Investigator would compromise safety or conduct of the study procedures.

Inclusion of Women and Minorities

Both men and women and members of all ethnic groups are eligible for this trial. Given the reduced incidence of melanoma in the non-white population, we expect that few of the subjects enrolled will be non-white.

4. Subject Selection and Enrollment

4.1. Identifying Participants

Eligible subjects will be those diagnosed with histologically confirmed, metastatic unresected stage lllb/c or lV melanoma for whom treatment with pembrolizumab is indicated. Subjects must have at least one cutaneous, subcutaneous tumor or palpable lymph node that is amenable to intratumoral injection. Subjects must meet all eligibility criteria as detailed above.

4.2. Consenting Participants

All subjects must receive, review and sign a Research Ethics Committee-approved informed consent form prior to any study-specific procedures or assessments being conducted. Consent of subjects must be conducted according to Good Clinical Practice. Only subjects who are capable of understanding and consenting to the trial will participate.

4.2.1 Subject Registration and Numbering

For Rutgers Cancer Institute only:

To register a subject, the investigator will call the Office of Human Research Services (OHRS) at the Rutgers Cancer Institute of New Jersey at PPD and speak to one of the nurse coordinators for the trial.

The following information will be requested:

- Investigator's name
- Subject's Identification
- Subject's name or initials and chart number
- Subject's Social Security number
- Eligibility Verification

For all other sites, the next available subject number will be used and the subject entered into the EDC system. If a subject enrolls in the study but does not receive study therapy, the subject's enrollment may be canceled. Reasons for cancellation will be documented in writing. Any subject whose enrollment was canceled before receiving study therapy will be replaced.

Study Numbers will be assigned at enrollment using the following formula: last 2-digits of the Viralytics protocol number + the 2-digit site number + the 3-digit subject number. For example:

- The first subject at site 06 would be 1106001

All case report forms, study reports, and laboratory samples for research tests, including immune parameters or pharmacokinetics, will be labeled with the full subject Study Number.

4.3. Screening for Eligibility

After written informed consent is obtained, demographic data, a complete medical and medication history, physical exam and laboratory samples will be obtained to confirm all eligibility criteria have been met. Subject eligibility screening procedures must be completed within 28 days prior to study treatment initiation (Day 1), but procedures to determine eligibility may be conducted over several days during this period. **Eligibility must be confirmed by the Viralytics Medical Monitor prior to randomization.**

4.4. Ineligible and Non-Recruited Subjects

Subjects who have signed an Informed Consent Form and who are subsequently deemed ineligible will be considered screen failures. If a subject is re-screened, the original screening number will be maintained. A new screen numbers will not be assigned to a subject previously screened.

4.5. Withdrawal Procedures

4.5.1 Study Treatment Discontinuation

All reasons for discontinuation of treatment must be documented. All subjects who are withdrawn from study treatment will be followed for survival or until death post-treatment.

Criteria for removal from study treatment are:

1. Symptomatic disease progression after CVA21 and pembrolizumab; the subject should be restaged and sites of recurrence and/or progression documented.
2. Unacceptable toxicity. Unacceptable toxicity is defined as:
   - any Grade ≥ 4 toxicity, or
   - any Grade ≥ 3 toxicity except constitutional symptoms that do not resolve to Grade 1 or better within 3 weeks.
   - Grade ≥ 3 laboratory abnormalities without clinical impact and adverse events that are not related to study drugs are excluded as indications to discontinue study treatment.
3. Subject's decision to withdraw from study treatment at any time for any reason.
4. Development of intercurrent, non-cancer related illnesses that prevent either continuation of therapy or regular protocol-defined visits.
5. Pregnancy

In the absence of symptomatic disease progression requiring alternate therapy or withdrawal of consent from the study, or withdrawal by the investigator, subjects may continue with protocol scheduled visits and will be followed for survival or until death post-treatment.

4.5.2 Study Discontinuation

Participation in this study is voluntary and subjects may be withdrawn from the study at any time without any loss of benefits to which they are otherwise entitled. For every subject a reason for discontinuation of the study is required. The following list provides possible reasons for discontinuation, in order of priority.

Reasons for discontinuation from the study include the following:

- Completed study – subject completed all visits to Day 358
- Death
- Lost to follow-up
- Investigator decision, after consultation with the Sponsor
- Subject Withdrawal of Consent
- Termination of study by Sponsor
- Other

Where possible, subjects who are withdrawn prior to the final visit must be brought in for an Early Termination visit, and visit procedures outlined in the Schedule of Procedures conducted with the exception of subjects who have withdrawn consent for further procedures, are lost to follow-up or in the case of death. Subjects who progress or who elect to discontinue tumor assessments will be followed for survival only, every 3 months for the first year, then every 6 months until discontinuation from the study.

5. Investigational Medicinal Product

5.1. CVA21

The investigational agent is a gradient-purified preparation of the Coxsackievirus A21 prototype strain consisting of infectious and non-infectious viral particles and free from mycoplasmal, viral and other contaminants of microbiological origin. The drug substance is produced in a cGMP-compliant environment. The investigational agent consists of drug substance diluted in 4% w/v sucrose in phosphate-buffered saline (PBS) at pH 7.2.

Storage: The drug product will be stored frozen at < -70°C in sealed glass vials.

Toxicity: The main toxicities of CVA21 are local injection site reactions including erythema and pain, low-grade temperatures, transient flu-like symptoms, arthralgias, and myalgias. To date, these toxicities have been mild or moderate in grade. Further information is available in the CVA21 Investigator's Brochure.

5.1.1 Dose Preparation

To load the syringe with CVA21, remove the vial from the individual carton and that at room temperature (18 – 25°C). Do not leave the vial at room temperature for longer than is necessary to thaw the contents. A lab coat, safety glasses, sterile gloves, and masks should be worn while loading the syringe with CVA21. Gently mix the vial for 5 seconds and tear off the plastic top. Use a luer-lock syringe of appropriate volume and 21-gaue needle to draw up the required volume. Remove air bubbles. Remove the withdrawal needle and replace with a 25-gauge capped needle. Hold on ice until required (2 – 8°C). Administer within 3 hours from loading the syringe distribute into the tumors as described in Appendix 15.4.

5.1.2 Dose Modifications

CVA21 administration will stop for any individual subject experiencing DLT from this agent. There will be no CVA21 dose modifications for individual subjects. If CVA21 dosing is stopped in an individual subject, any remaining planned pembrolizumab doses can be administered if clinically indicated. For subjects experiencing pembrolizumab toxicity, these toxicities will be managed per established guidelines (included in Appendix 15.3). http://www.merck.com/product/usa/pi. If pembrolizumab dosing is stopped, any remaining CVA21 doses may be given. The general strategy to abrogate immune-mediated toxicities is to administer corticosteroids (e.g., prednisone) until the toxicity resolves, followed by a taper of the steroid dose. If the toxicity does not recur during the taper and no other adverse events ensue, then pembrolizumab dosing can continue.

For non-treatment related toxicities that may warrant holding one or both study drugs, dosing will resume as normally scheduled at the next scheduled visit assuming toxicities have resolved to an appropriate level.

5.1.3 Administration

The drug will be supplied by Viralytics. Each subject will receive CVA21 up to a total dose of 3 x10^8 $TCID_{50}$ (about 4.5 x 10^6 $TCID_{50}$/kg for a 70-kg subject) in a maximum volume of 4.0 mL by intratumoral administration on Days 1, 3, 5, 8 and at 3-weekly intervals (up to a maximum of 19 total injections) until confirmed disease progression, complete response or development of excessive toxicity. At each scheduled injection visit, if possible, multiple lesions, are injected in a dose hyper-fraction pattern, starting with the largest lesion(s) to a maximum of 4.0 mL using Table 2 to determine the volume to be injected.

The largest diameter of each tumor to be injected is measured and the volume of CVA21 to be injected into each tumor determined. Tumors may be measured by ruler/calipers or using calipers on ultrasound. The sum of these volumes is the total volume of CVA21 required for the administration. The maximum volume of CVA21 to be administered is 4.0mL. Following the initial injection with CVA21, any injected lesion that reduces in diameter to <5mm will be injected with 0.1mLs of CVA21 per the stated treatment schedule until the lesion completely resolves.

The following guidelines should be followed regarding order of preference for lesions to be injected, if 4.0mLs is determined to be insufficient to inject all qualifying lesions:

1. Lesions injected at baseline, both index and non-index
2. Index lesions that were present at baseline but were not previously injected
3. New lesions not present at baseline that are ≥5mm for non-lymph node (LN) lesions and ≥ 15mm for LN (short axis diameter)

Any newly injected lesions should continue to be injected for the remainder of the study, subject to the order of preference above and without exceeding the maximum 4mL dose.

Tumors to be injected lying in mucosal regions or close to an airway, major blood vessel or spinal cord that, in the opinion of the Investigators, could cause occlusion or compression in the case of tumor swelling or erosion into a major vessel in the case of necrosis are NOT to be injected with CVA21.

Table 2. CVA21 Administration Volume by Tumor Diameter

Tumor Diameter	Volume of CVA21
> 25 mm	2.0 mL
15 – 25 mm	1.0 mL
5 < 15 mm	0.5 mL

Tumor Diameter	Volume of CVA21
< 5 mm*	0.1 mL

* only for lesions previously treated with CVA21

Following CVA21 administration, injections sites should be wiped with sterile tissue and an occlusive dressing applied to completely cover the injection site. Used tissues should be placed in appropriate infectious waste containers. In laboratory studies, the major side effect toxicity observed in mice injected with CVA21 is the development of hind-limb paralysis resulting from inflammation in the muscles of the leg, in particular following intramuscular injection. However, in the clinical experience of Viralytics to date, no signs of the development of myositis have been observed in human subjects administered CVA21 by either intratumoral or intravenous routes. Therefore, CVA21 is **NOT** to be administered via the intramuscular route in this study.

Any unused vials will be returned to Viralytics or destroyed on site per institutional standard operating procedures following written authorization from Viralytics.

Disposal of Contaminated Materials and Management of Accidental Spills:

Viral administration should be performed in a manner to minimize potential exposure to other cancer suffering or immunocompromised individuals. Furthermore, systematic decontamination of surfaces that may directly come into contact with viral inoculum or excretion samples must be undertaken, with an appropriate anti-viral agent (e.g., Virkon®, a sodium hypochlorite solution [5-6%] or formaldehyde [3%]).

All materials utilized in the viral administration must be disposed of in appropriate infectious waste containers. Any mishap such as accidental spillage or inoculation might expose a staff member to virus in ways not occurring naturally, although there is nothing to suggest they would be at particular risk as a result. Nevertheless, because of the above, it would be advisable that handling and inoculation of virus be done by staff trained in handling infectious agents and disposal of potentially infectious waste. Accidental spills from samples and operations should be adsorbed with paper tissues soaked with an appropriate anti-viral agent (e.g., Virkon®, a sodium hypochlorite solution [5-6%] or formaldehyde [3%]) and then with water.

Tissues should then be discarded in proper containers designated for infectious laboratory/hospital waste.

In addition, any institutional biosafety standard operating procedures should be implemented during virus handling, administration and in the event of a spill or accidental exposure.

5.1.4 Intra-Subject Dose-Escalation

All subjects will receive the same dose of CVA21; no intra-subject dose escalation will occur. Dose escalations will not be permitted. Standard supportive medications including antiemetics and pain medications will be offered during treatment. Steroids will not be used for the treatment of nausea, but can be used to ameliorate pembrolizumab-induced immune related toxicity as detailed in Appendix 15.3.

5.2. Pembrolizumab

Please see the full package insert in Appendix 15.3. Pembrolizumab (Keytruda ™) is a humanized monoclonal antibody against the programmed death receptor-1 (PD-1) protein and has been developed by Merck and Co for the treatment of cancer. Pembrolizumab is an IgG1 kappa immunoglobulin with an approximate molecular weight of 149 kDa. Pembrolizumab is commercially available and will not be provided by the Sponsor.

Pembrolizumab is a humanized monoclonal antibody that blocks the interaction between PD-1 and its ligands, PD-L1 and PD-L2. Pembrolizumab is an IgG4 kappa immunoglobulin with an approximate molecular weight of 149 kDa.

Toxicity: Side effects of pembrolizumab are fatigue, cough, nausea, pruritus, rash, decreased appetite, constipation, arthralgia, and diarrhea. Appendix 15.3 contains information for management of Pembrolizumab.

How Supplied: Pembrolizumab is a sterile, preservative-free, white to off-white lyophilized powder in single-use vials. Each vial is reconstituted and diluted for intravenous infusion. Each 2 mL of reconstituted solution contains 50 mg of pembrolizumab and is formulated in L-histidine (3.1 mg), polysorbate-80 (0.4 mg), sucrose (140 mg). May contain hydrochloric acid/sodium hydroxide to adjust pH to 5.5.

Storage: The product does not contain a preservative. Do not freeze.

Store the reconstituted and diluted solutions of pembrolizumab either:

- At room temperature for no more than 4 hours from the time of reconstitution. This includes room temperature storage of reconstituted vials, storage of the infusion solution in the IV bag, and the duration of infusion.
- Under refrigeration at 2°C to 8°C (36°F to 46°F) for no more than 24 hours from the time of reconstitution. If refrigerated, allow the diluted solution to come to room temperature prior to administration.

5.2.1 Dose Preparation

1. Add 2.3 mL of Sterile Water for Injection, USP by injecting the water along the walls of the vial and not directly on the lyophilized powder (resulting concentration 25 mg/mL).
2. Slowly swirl the vial. Allow up to 5 minutes for the bubbles to clear. Do not shake the vial.
3. Visually inspect the reconstituted solution for pariculate matter and discoloration prior to administration. Reconstituted pembrolizumab is a clear to slightly opalescent, colorless to slightly yellow solution. Discard reconstituted vial if extraneous particulate matter other than translucent to white proteinaceous particles is observed.
4. Withdraw the required volume from the vial(s) of pembrolizumab and transfer into an intravenous (IV) bag containing 0.9% Sodium Chloride Injection, USP. Mix diluted solution by gentle inversion. The final concentration of the diluted solution should be between 1 mg/mL to 10 mg/mL.
5. Discard any unused portion left in the vial.

5.2.2 Dose Modifications

See section 5.1.4 for complete dose modification instructions. In this study, for pembrolizumab, guidelines for administration and toxicity management as defined in the Keytruda ™ Package Insert (Appendix 15.3) will be followed, which are in accordance with the FDA labeling for this medication. In addition to the guidelines for toxicity in the package insert, pembrolizumab may be withheld for grade 1 or grade 2 intolerable toxicity, if it is determined to be in the best interest of the subject, in consultation with the Viralytics Medical Monitor.

5.2.3 Administration

Administer infusion solution intravenously over 30 minutes through an intravenous line containing a sterile, non-pyrogenic, low-protein binding 0.2 micron to 5 micron in-line or add-on filter. Do not co-administer other drugs through the same infusion line. On days when pembrolizumab and CVA21 are both administered, pembrolizumab is administered after CVA21.

5.2.4 Intra-Subject Dose Escalation

See section 5.1.4 for complete dose escalation instructions.

5.3. Dose-limiting Toxicity

Toxicities will be assessed using CTCAE v. 4.03. Dose-limiting toxicity (DLT) is defined as any grade ≥ 3 toxicities believed related or possibly related to CVA21, with onset on or before the Day 92 visit, with the exception of lymphopenia, which will not be considered a DLT. If a CVA21-related DLT is observed in 2 subjects among the first 6 treated, then the study will be terminated. If no DLTs are encountered or if the proportion of CVA21-related grade ≥ 3 toxicity is less than 30% of the subjects accrued, then the study accrual may continue. The toxicity will be independently monitored by Data Monitoring Committee which will communicate any safety concerns directly to all participating Investigators.

5.4. Subject Compliance

Study drug compliance is not expected to an issue as CVA21 and pembrolizumab are administered by site personnel during study visits.

5.5. Overdose

Should a subject receive a dose in excess of that allowed, the Medical Monitor must be notified immediately. Any overdose should be documented in the Pharmacy records and eCRF and recorded as a protocol deviation.

5.6. Prior, Concomitant and Subsequent Therapy

With the exception of prohibited medications, treatment that the investigator considers necessary for a subject's welfare may be administered at the discretion of the investigator in keeping with the community standards of care and GCP. All concomitant medication will be recorded on the eCRF including all prescription, over-the-counter (OTC), herbal supplements, and IV medications. All changes that occur during the trial period will be recorded in the eCRF.

All concomitant medications received within 30 days of the first dose of either study drug and 30 days after the last dose of both study drugs should be recorded.

Prohibited Treatment:

Subjects are prohibited from receiving the following therapies during the Screening and Treatment periods of this study:

- Antineoplastic systemic chemotherapy or biological therapy
- Immunotherapy not specified in the protocol
- Chemotherapy not specified in the protocol
- Other investigational agents

Systemic steroids are prohibited unless indicated for the treatment of autoimmune-related adverse events. Intranasal, intraarticular, and inhaled steroids are allowed. Consult the Viralytics Medical Monitor for confirmation.

5.7. Diet/Pregnancy/Contraception/Other Considerations

Diet: Subjects should maintain a normal diet unless modifications are required to manage AE such as diarrhea, nausea or vomiting.

Pregnancy: Throughout the study and for at least 4 weeks following the last administration of study drug, women of child-bearing potential should consistently and correctly use a highly effective method of contraception such as implants, injectable, combined oral contraceptives, some intrauterine devices (IUD), barrier methods, sexual abstinence or vasectomized partner.

Male subjects with partners who are pregnant or of child-bearing potential, should practice sexual abstinence or use consistently and correctly a male condom for the duration of the study and for at least 4 weeks following the last administration of study drug. Furthermore, it is recommended that sperm donations are not to be undertaken during the study and for at least 4 weeks following the last administration of study drug.

5.8. Dispensing and Accountability

The investigator shall take responsibility for and shall take all steps to maintain appropriate records and ensure appropriate supply, storage, handling, distribution and usage of trial treatments in accordance with the protocol and any applicable laws and regulations.

The Investigator will maintain a log of all CVA21 and pembrolizumab shipments including, but not limited to, date of shipment, number of vials received, lot number, date dispensed, subject dispensed to and current inventory. Accountability logs will be provided by the Sponsor and reviewed by the Sponsor's designated Monitor periodically during the study.

Refer to the Pharmacy Manual for detailed procedures.

6. Study Assessments

6.1. Safety Assessments

Clinical safety assessments will be performed at study visits per the Schedule of Procedures (Appendix 15.1)

6.1.1 Physical Examination

Physical examinations will include examination of general appearance, skin, neck (including thyroid), eyes, ears, nose, throat, heart, lungs, abdomen, lymph nodes, extremities and nervous system. Full physical exam will be conducted at Screening and Study Day 1. All other visits will require a symptom-directed physical examination. An AE CRF must be completed for all clinically noteworthy changes identified.

6.1.2 Vital Signs and Body Weight

Height and weight without shoes will be recorded in centimeters and kilograms respectively. Vital signs will include blood pressure, heart rate, respiratory rate and temperature.

6.1.3 ECG

Standard 12-lead electrocardiogram (ECG) will be conducted and will include calculation of QTc Interval.

6.1.4 Brain MRI

Standard brain MRI is to be conducted at screening as clinically indicated. Brain MRI may be repeated as clinically indicated.

6.1.5 Laboratory Parameters

The following laboratory tests are to be performed as indicated by the Schedule of Procedures:

**Assessments for safety and tolerability**		
**Hematology**	**Serum Chemistry**	**Hormones**
Red blood cells<br>White blood cells (WBCs)<br>Hemoglobin<br>Hematocrit<br>Platelet count<br><br>Differential WBC Count:<br>Absolute neutrophil count<br>Absolute lymphocyte count<br>Absolute monocyte count<br>Absolute eosinophil count<br>Absolute basophil count<br><br>PT<br>PTT	Alanine aminotransferase (ALT/SGPT)<br>Aspartate aminotransferase (AST/SGOT)<br>Alkaline phosphatase<br>Total bilirubin<br>Glucose<br>Albumin<br>Total protein<br>Creatinine<br>Blood urea nitrogen<br>Chloride<br>Potassium<br>Sodium<br>Bicarbonate<br>Calcium<br>Lactate dehydrogenase<br>Serum pregnancy<br>**Thyroid Panel**: TSH, Free T4, T3 or Free T3.	Cortisol<br>ACTH

Local Lab: Laboratory samples will be analyzed by local laboratories for interpretation of results. In the event of an unexplained clinically noteworthy abnormal laboratory test value, the test should be repeated immediately and followed up until it has returned to the normal range and/or an adequate explanation of the abnormality is found.

Central lab: In addition to the above, serum samples drawn at the same time as the local laboratory samples (prior to administration of CVA21 or pembrolizumab) will be forwarded to the Central Laboratory for assessing neutralizing antibodies to CVA21 and sICAM-1 and for assessing CVA21 serum load. Refer to the Laboratory Manual for sample processing details.

6.1.6 Pregnancy Test

Female subjects of child-bearing potential must have a negative serum pregnancy test within 72 hours prior to receiving the first dose of study medication and as indicated by the Schedule of Procedures.

6.1.7 CVA21 Excretion Testing

Excretion testing is not being conducted under this protocol.

6.1.8 CVA21 Transmission Precautions and Procedures

Subject and Care Giver Precautions

There is a possibility that subjects in the study may excrete virus from the respiratory or gastrointestinal tract for some days after CVA21 administration. As such, caregivers, family members, clinical trial staff, and healthcare workers must be aware of this potential shedding and follow the guidance listed below. However, it must be noted that potential shedding is unlikely to be quantitatively different from naturally acquired infection.

Primary caregivers should be fully advised that the subject in the study may excrete infectious virus in respiratory and gastrointestinal tract secretions for a number of weeks following the initial viral injection. The caregivers should be fully informed that CVA21 is acquired naturally by the respiratory route and circulates naturally in the community from time to time. Precautions that are normally undertaken during the general prevention of respiratory infections should be followed. Hands should be washed with soap and warm water immediately following handling of feces/fecal soiled clothing, and material exposed to respiratory secretions such as tissues and handkerchiefs. Caregivers and family members should observe personal hygiene (i.e., hand washing) following contact with recently treated subjects. Caregivers who present symptoms of a cold or flu should present to a study investigator to provide a sample for analysis of the underlying infection.

Should a subject develop symptoms of a suspected respiratory infection, they should wear a mask when attending study visits to minimize any risk of aerosol generation during coughing.

Procedural Precautions

CVA21 is classified as Biosafety Level 2 (BSL-2) biohazard material and all precautions and procedures for BSL-2 laboratory should be observed when handling CVA21 or the contaminated equipment and objects that have been in contact with CVA21. Further information may be found in the Pharmacy Manual.

All waste generated during the dispensing and administration of CVA21 (including sharps, needles, and vials as indicated) that have been in contact with the virus will be placed in a biohazard bag or sharps bin and incinerated. During clean-up, protective clothing, properly fastened, must be worn by all personnel.

Eye and face protection, including surgical masks, must be worn when applicable to guard against any splashing or aerosol droplets. Safety glasses must be decontaminated by wiping all surfaces with disinfectant (i.e. Virkon). Gloves must be worn for all procedures that might involve direct skin contact and hands must be washed after gloves have been removed.

All equipment and work surfaces must be decontaminated with disinfectant (i.e. Virkon, 3% formaldehyde or 5-6% sodium hypochlorite).

All protective clothing must be removed before going beyond the contaminated areas and will be placed in a biohazard bag. If items are non-disposable they will then be laundered according to normal hospital policy for bio-hazardous items. Disposable items must be incinerated.

Empty or partially filled vials of CVA21 should be disposed of per local procedures after preparing the study drug administration syringes, with the empty carton kept for monitoring drug accountability.

The Sponsor recommends that during the course of the study all staff involved in the viral administration and the collection of excretion samples wear protective gloves and face masks that are disposed of immediately. Although CVA21 is primarily transmitted via aerosols, CVA21 injection sites should be covered with an occlusive dressing that covers the entire lesion. This dressing should remain in place until the next scheduled visit, when it will be replaced by a new dressing until the site is dry and clear. Should a dressing be dislodged, a new one should be applied and the old one disposed of in appropriate infectious waste containers.

Viral administration should be performed in a manner to minimize potential exposure to immunocompromised individuals, i.e., viral administration should be performed at the end of a scheduled clinic or in a room distanced from or annexed to the major subject treatment room. While there is no requirement to decontaminate the bed or room where the injection was performed, systematic decontamination of surfaces that may have come into direct contact with CVA21, subject blood or excretion samples must be undertaken with an appropriate antiviral agent (e.g., Virkon® a sodium hypochlorite solution [5-6%], or formaldehyde [3%]). All materials utilized in the viral administration must be disposed of in appropriate infectious waste containers. Please refer to the CVA21 Material Safety Data Sheet for further details.

Staff injury by needles that have been in contact with the virus should be treated as per the standard occupational health guidelines on needle injuries. Staff should inform the occupational health department and both the principal investigator and research nurse involved in the trial of the injury. Any member of staff involved in direct subject care and/or sample handling who becomes unwell should report the illness to the occupational health department, principal investigator and research nurse.

Procedures for Suspected Cases of Transmission

Any mishap such as accidental spillage or inoculation might expose a staff member to virus in ways not occurring naturally, although there is nothing to suggest they would be at particular risk as a result. Nevertheless, because of the above, it would be advisable that handling and inoculation of virus be done by staff trained in handling infectious agents and disposal of potentially infectious waste.

If a healthcare worker or close contact reports illness that is possible from viral transmission of the injected Subjects, the back of the throat of the healthcare worker or close contact should be swabbed with a sterile cotton swab near the tonsils. The subject should resist gagging and closing the mouth while the swab touches the back of the throat. To improve the chances of detecting infectious agents, the swab may be used to scrape the back of the throat several times. The swab should be placed immediately in viral transport media (e.g., Universal viral transport media, Becton Dickinson # 220221) and the sample stored at 4°C until transportation to the testing laboratory. The close contact or healthcare person should wash their hands immediately. In addition or in place of the throat swab, a sputum sample may be given from the close contact or healthcare person. The close contact or healthcare person subject will be asked to cough deeply and expectorate any material that comes up from their lungs into a special collection container and the sample kept at 4°C until transportation to the testing laboratory. The subject or healthcare person should wash his/her hands immediately.

Please see the procedures in Appendices 15.8 and 15.10 for further details.

6.2. Efficacy Assessments

6.2.1 Imaging

Disease status will be assessed by computerized tomography (CT) or magnetic resonance imaging (MRI) scans and other appropriate measures (i.e., Physical Exam measurement) every 6 weeks. Response at these visits will be assessed according to immune-related response criteria (Appendix 15.6.)

6.2.2 Photography

Photographs (optional) are to be taken of all visible lesions (following subject consent) at Screening and Days 50, 113, 197, 281, 365, 449, 533, 617 and 701 as described in the Schedule of Procedures. Refer to the Photography Manual for further details.

6.2.3 QOL/FACT-BRM

A commonly used quality of life (QOL) questionnaire know as FACT-BRM (Functional Assessment of Cancer – Biological Response Modifier) shall be used before and during the planned study treatment (Appendix 15.12). This survey tool was designed for subjects with melanoma specifically and will be administered prior to performing study procedures at Screening and Days 50, 113, 197, 281, 365, 449, 533, 617 and 701 as described in the Schedule of Procedures (Appendix 15.1)

6.3. Exploratory Assessments

Immunological Monitoring (selected sites only)

As detailed in the study schema, samples for immunological monitoring will include optional melanoma tumor biopsies, and collecting peripheral blood mononuclear cells (PBMC). The main

objectives of the monitoring will be to characterize circulating T-cell subsets and antibody responses to representative antigens found on melanoma in addition to an assessment of tumor PD-L1 expression.

The immunological monitoring lab will perform flow cytometry with a panel of markers including CD3, CD4, CD8, CD27, CD28, CD95, CD25, CD127, CCR-7, FoxP3, ICOS, and CD45RA. T cell subpopulations of interest include Treg, central memory and effector cells. Other immunological measures may be evaluated including but not limited to immunoscore of biopsy samples, assessment of melanoma-specific tumor responses using overlapping peptide libraries and ELISPOT, measurement of serum cytokine levels using Luminex beads and anti- Coxsackievirus A21 antibody development to generate hypotheses for future studies and to gain preliminary information on possible biomarkers.

T-cell response to melanoma cell lines and antigens identified by protein array may also be analyzed using autologous tumor if available from biopsy or using a bank of melanoma specimens. Assessment will start by evaluating whether post treatment samples recognize HLA-matched melanoma cells. If subjects are identified with detectable interferon gamma responses against matched melanoma cells evaluation of whether the same response exists in the limited number of pre-treatment cryopreserved PBMC will be performed.

Tumors selected for (optional) biopsy must be anatomically accessible by clinical exam or ultrasound. Methods of biopsy can include incisional, excisional, or punch biopsy. Tumors selected may be non-index lesions, or index lesions. Index lesions selected for biopsy must be greater than 20 mm in total size and care should be taken to take a biopsy small enough so as not to affect their longest diameter for purposes of tumor assessment.

PBMC will be collected at baseline (Screening or Day 1) and prior to pembrolizumab administration for doses 2, 4 and 6 and at study termination. Other collection timepoints indicated on the Study Calendar (Section 6) are optional. Collection will include 8 green top and 2 red top tubes for PBMC and serum. All samples will be collected and processed in the Tumor Immunology Laboratory:

PPD

6.4. Study Assessments (Visit by Visit Procedures)

Refer to the Schedule of Procedures (Appendix 15.1) for visit by visit procedures.

7. Data Collection

7.1. Data Collection and Retrieval

Clinical data will be recorded on study-specific, password-protected and secure electronic case report forms (CRFs) provided by the CRO. All data entries to CRFs must be supported by a clinical source document. No direct entry of subject data to the CRF is permitted.

Study site staff responsible for data entry will be assigned password-protected user-specific access once they have been trained in the use of the eCRF system.

7.2. Investigator Reporting Requirements

The Investigator is responsible for the quality of the data recorded in the eCRF. Data should be entered within 72 hours of the subject's visit.

7.3. Record Retention

All study documentation will be kept by the site for at least 15 years after publication of the study data.

8. Statistics and Data Analysis

8.1. Sample Size Calculation

A two-stage design often used for phase II studies will be applied with the objective of estimating response rate. Simultaneously, as a phase I trial, safety of CVA21 will be assessed and the study stopped early for subject safety according to stopping rules defined in Section 4.5.2. The reported toxicity rate for CVA21 is low. There were no grade ≥ 3 toxicities observed in 57 subjects treated in the VLA-007 study. The toxicity rate for the combined therapy is expected to be comparable to that for pembrolizumab alone.

Simon's two-stage design will be used.[32] The null hypothesis that the true response rate (CR+PR) is 0.28 will be tested against a one-sided alternative. In the first stage, 18 subjects will be accrued. If there are 5 or fewer responses in these 18 subjects within 12 months of starting treatment, the study will be stopped due to futility of treatment. Otherwise, a minimum of 27 additional subjects will be accrued for a total of 45. The null hypothesis will be rejected if 18 or more responses are observed in 45 subjects within 12 months of starting treatment. This design yields an α =0.05 level of significance with a power of 90% to detect a difference when the true response rate is 0.50. Historical response rate (CR+PR) of 0.28 is based on response rate reported for CVA21 monotherapy in the VLA-007 study. An objective response rate of 0.28 in ipilimumab-pretreated subjects following pembrolizumab treatment was reported from the KEYNOTE-001 study (NCT01295827).

The sample size of 45 subjects may be increased by up to 5 additional subjects to adjust for subjects who do not complete the Day 113 lesions assessment. Thus, the total sample size is estimated to be 50 subjects.

8.2. Data Analysis

A Statistical Analysis Plan (SAP) will provide the details for all summaries and analyses to be provided in the clinical study report for this study. The Safety population is defined as all subjects who have been enrolled and received at least one dose of CVA21 and/or pembrolizumab. This population is the primary population for analysis of safety and efficacy.

8.2.1 Disposition, Demographics and Baseline Characteristics

Summaries of disposition, demographics, and baseline data will be provided.

8.2.2 Efficacy Data

The best response for each subject based on irRC criteria will be summarized for each cohort and overall. Overall response rate (ORR), defined as a best response of partial response (PR) or better will also be summarized. Two-sided exact 90% and 95% CI will be constructed for ORR.

Durable response rate (DRR) will be defined as the percentage of subjects demonstrating a best overall response of irCR or irPR lasting at least 26 weeks (using irRC). DRR along with the associated exact binomial confidence intervals will be determined. Subjects lacking valid data to assign a response status will be classified as non-responders.

Progression-free survival (PFS) is defined as the time from the beginning of treatment to PD or death, whichever occurs first. Duration of response is defined for subjects with a best overall response of irPR or irCR as the time from first documentation of response to PD or death, whichever occurs first. Summary statistics for PFS and duration of response will be provided using Kaplan-Meier methods.

Rules for handling missing or partial dates and censoring rules for PFS and duration of response will be specified in the SAP.

Exploratory measures of immune activation in tumor tissue will be summarized. Additional exploratory analyses may be performed.

The QOL data (using FACT-BRM) will be summarized at each scheduled time point.

8.2.3 Safety Data

Safety assessments include AEs, laboratory values, and vital signs. The baseline value of laboratory data and vital signs is the most recent value recorded on or before the first day of study treatment.

Study drug exposure, including duration, total dose, and dose modifications will be summarized. Treatment-emergent adverse events (TEAEs) are defined as AEs that start on or after the first day of study treatment and within 30 days of the last administration of study treatment. The incidence of TEAEs will be summarized based on the number and percentage of subjects in each cohort and overall who experience events classified by MedDRA system organ class and preferred term. In the event that a subject experiences repeated episodes of the same AE, the subject will be counted once within each system organ class and once within each preferred term. Summaries of the incidence of treatment-related AEs, serious AEs, and AEs classified according to severity grade will be provided. The event with the highest severity grade and/or strongest causal relationship to treatment will be used for these summaries.

Laboratory data results (actual value and change from baseline) for selected hematology chemistry tests will be summarized for each cohort and overall at each scheduled time point. Subjects with missing data for a given time point will not contribute to the summary for that time point. Laboratory results from samples taken > 30 days after the last administration of study treatment will be excluded from these summaries.

Selected laboratory test results will be assigned toxicity grades using the National Cancer Institute's CTCAE. Shifts in severity grades for selected laboratory tests will be summarized for each cohort by comparing the maximum toxicity grade observed after the start of study treatment to the baseline toxicity grade. All available laboratory results will be included in these summaries. All serum chemistry and hematology results (actual value and change from baseline) will be summarized at each scheduled time point.

Vital sign results (actual value and change from baseline) will be summarized for each cohort and overall at each scheduled time point.

9. Adverse Events

The Investigator is responsible for the detection and documentation of events meeting the criteria and definitions detailed below.

Full details of contraindications and side effects that have been reported following administration of the trial drug can be found in the Investigator's Brochure.

Participants should be instructed to contact their Investigator at any time after consenting to join the trial if any symptoms develop. All reported adverse events (AEs) that occur after joining the trial must be recorded in detail in the CRF. In the case of an AE, the Investigator should initiate the appropriate treatment according to their medical judgment. Participants with AEs present at the last visit must be followed up until resolution or stabilization of the event.

Progression of the cancer under study is not considered an adverse event unless it is considered to be drug-related by the investigator.

All adverse events that occur after the consent form is signed but before treatment initiation (Day 1) must be reported by the investigator if they cause the subject to be excluded from the trial, or are the result of a protocol-specified intervention, including but not limited to washout or discontinuation of usual therapy, diet, placebo treatment or a procedure.

From the time of treatment initiation (Day 1) through 30 days following cessation of treatment, all adverse events must be reported by the investigator. Such events will be recorded at each examination on the Adverse Event case report forms/worksheets. The reporting timeframe for adverse events meeting any serious criteria is described in section Immediate Reporting of Adverse Events to the Sponsor. The investigator will make every attempt to follow all subjects with non-serious adverse events for outcome.

9.1. Definitions

An **adverse event** (AE) is any untoward medical event affecting a clinical trial participant. Each initial AE will be considered for severity, causality or expectedness and may be reclassified as a serious event or reaction based on prevailing circumstances

An **adverse reaction (AR)** is where it is suspected that an AE has been caused by a reaction to a trial drug.

A **serious adverse event** (SAE), **serious adverse reaction** (SAR) or **suspected unexpected serious adverse reaction** (SUSAR) is any AE, AR or UAR that at any dose:

- results in death;
- is life threatening (i.e. the subject was at risk of death at the time of the event; it does not refer to an event which hypothetically might have caused death if it were more severe);
- requires hospitalization or prolongation of existing hospitalization;
- results in persistent or significant disability or incapacity;
- is a congenital anomaly or birth defect.

Note: Hospitalizations for treatment planned prior to randomization and hospitalization for elective treatment of a pre-existing condition will not be considered as an AE. Complications occurring during such hospitalization will be AEs or SAEs as appropriate.

Unexpected adverse event:

Any adverse drug experience, the specificity or severity of which is not consistent with the current investigator brochure; or, if an investigator brochure is not required or available, the specificity or severity of which is not consistent with the risk information described in the general investigational plan or elsewhere in the current application, as amended.

Associated with the use of the drug/intervention:

There is a reasonable possibility that the experience may have been caused by the drug.

Disability:

A substantial disruption of a person's ability to conduct normal life functions.

Life-threatening adverse event:

Any adverse drug experience that places the subject, in the view of the investigator, at immediate risk of death from the reaction as it occurred, i.e., it does not include a reaction that, had it occurred in a more severe form, might have caused death.

Unanticipated Problem:

An unanticipated problem is an adverse event that is (i) unexpected; (ii) serious; and (iii) felt by the investigator to be possibly, probably, or definitely related to the research intervention. Only adverse events that meet this definition need be reported to the IRB.

For more information on the definition of an unanticipated problem and reporting requirements, consult the current Investigator's Brochure.

9.2. Reporting Overdoses

All overdose should be reported to the Sponsor and Medical Monitor as soon as the overdose is noted.

In the event of an overdose, the subject should be observed closely for signs of toxicity. Appropriate supportive treatment should be provided if clinically indicated.

9.3. Reporting of Pregnancy

Although pregnancy is not considered an adverse event, it is the responsibility of investigators or their designees to report any pregnancy in a study subject (spontaneously reported to them) that occurs during the trial.

Pregnancies that occur after the consent form is signed but before treatment must be reported by the investigator if they cause the subject to be excluded from the trial.

Pregnancies that occur from the time of enrollment through 120 days following cessation of study treatment, or 30 days following cessation of treatment if the subject initiates new anticancer therapy, must be reported by the investigator. All reported pregnancies must be followed to completion/termination of the pregnancy.

Such events must be reported within 24 hours to the Sponsor. The site will contact the subject at least monthly and document the subject's status until the pregnancy has been completed or terminated.

9.4. Recording AEs and SAEs

When an AE/SAE occurs, it is the responsibility of the Investigator to review all documentation (e.g., hospital notes, laboratory and diagnostic reports) related to the event. The Investigator should then record all relevant information in the CRF and on the SAE form.

Information to be collected includes dose, type of event, onset date, Investigator assessment of severity and causality, date of resolution as well as treatment required, investigations needed and outcome.

9.5. Evaluation of AEs an dSAEs

Seriousness, causality, severity and expectedness should be evaluated as though the participant is taking active drug.

9.5.1 Assessment of Seriousness

The Investigator should make an assessment of seriousness as defined in **Section 9.1**.

9.5.2 Assessment of Causality

The Investigator must make an assessment of whether the AE/SAE is likely to be related to treatment according to the following definitions:

Unrelated: This category applies to those AEs that are clearly and incontrovertibly due to extraneous causes (disease, environment, etc.).

Unlikely: This category applies to those AEs that are judged to be unrelated to the test drug, but for which no extraneous cause may be found. An AE may be considered unlikely to be related to study medication when it meets two of the following criteria: (1) it does not follow a reasonable temporal sequence from administration of the test drug; (2) it could readily have been produced by the subject's clinical state, environmental or toxic factors, or other modes of therapy

administered to the subject; (3) it does not follow a known pattern of response to the test drug; or (4) it does not reappear or worsen when the drug is re-administered.

Possibly: This category applies to those AEs for which a connection with the test drug administration appears unlikely but cannot be ruled out with certainty. An AE may be considered possibly related when it meets two of the following criteria: (1) it follows a reasonable temporal sequence from administration of the drug; (2) it could not readily have been produced by the subject's clinical state, environmental or toxic factors, or other modes of therapy administered to the subject; or (3) it follows a known pattern of response to the test drug.

Probably: This category applies to those AEs that the investigator feels with a high degree of certainty are related to the test drug. An AE may be considered probably related when it meets 3 of the following criteria: (1) it follows a reasonable temporal sequence from administration of the drug; (2) it could not be reasonably explained by the known characteristics of the subject's clinical state, environmental or toxic factors, or other modes of therapy administered to the subject; (3) it disappears or decreases on cessation or reduction in dose (note that there are exceptions when an AE does not disappear upon discontinuation of the drug, yet drug-relatedness clearly exists; for example, as in bone marrow depression, fixed drug eruptions, or tardive dyskinesia); or (4) it follows a known pattern of response to the test drug.

Definitely: This category applies to those AEs that the investigator feels are incontrovertibly related to test drug. An AE may be assigned an attribution of definitely related when it meets all of the following criteria: (1) it follows a reasonable temporal sequence from administration of the drug; (2) it could not be reasonably explained by the known characteristics of the subject's clinical state, environmental or toxic factors, or other modes of therapy administered to the subject; (3) it disappears or decreases on cessation or reduction in dose and recurs with re-exposure to drug (if rechallenge occurs); and (4) it follows a known pattern of response to the test drug.

All AEs/SAEs judged as having a reasonable suspected causal relationship (e.g., possibly, probably, definitely) to the study drug will be considered as ARs/SARs. All AEs/SAEs judged as being related (e.g., possibly, probably, definitely) to an interaction between the study drug and another drug will also be considered to be ARs/SAR.

Alternative causes such as natural history of the underlying disease, concomitant therapy, other risk factors and the temporal relationship of the event to the treatment should be considered. The blind should not be broken for the purpose of making this assessment.

9.5.3 Assessment of Severity

The severity of AEs will be assessed according to the NCI CTCAE, version 4.03. If the AE is not defined in the CTCAE, the Investigator will determine the severity based on the following definitions:

- **Mild (Grade 1):** asymptomatic or mild symptoms; clinical or diagnostic observations only; intervention not indicated.
- **Moderate (Grade 2):** minimal, local or non-invasive intervention indicated; limiting age-appropriate instrumental ADL.*
- **Severe (Grade 3):** medically significant but not immediately life-threatening;

hospitalization or prolongation of hospitalization indicated; disabling; limiting self-care ADL.**

- **Life-threatening (Grade 4):** urgent intervention indicated.
- **Death (Grade 5):** death related to AE.

* Instrumental ADL refer to preparing meals, shopping for groceries or clothes, using the telephone, managing money, etc.

** *Self-care ADL refer to bathing, dressing and undressing, feeding self, using the toilet, taking medications, and not bedridden.*

9.5.4 Assessment of Expectedness

If an event is judged to be an AR/SAR, the evaluation of expectedness should be made based on knowledge of the reaction and the relevant product information documented in the SmPC and Investigator's Brochure.

9.6. Reporting of SAE/SARs and SUSARs

Once the Investigator becomes aware that an SAE has occurred in a study participant, they must report the information to the Sponsor within 24 hours of becoming aware of the event.

The Investigator must complete and submit the Adverse Event Form and Complementary Page for SAEs in the Electronic Data Capture (EDC) system. The EDC system will be the primary method used for SAE reporting to Precision Oncology Drug Safety and every attempt should be made by the Investigator or designee to enter all SAE data into the system. In the event the EDC system is not operational then the Paper SAE Report Form must be filled out and faxed to Precision Oncology Drug Safety. All SAE reports that have the minimum data set for reporting should be submitted via the EDC system, even if only limited information is available. This must occur within 24 hours of discovery of the event regardless of the relationship (or lack thereof) of the SAE to the CVA21 or pembrolizumab. If all the required information is not available at the time of reporting, the Investigator must ensure that any missing information is provided as soon as this becomes available. It should be indicated on the report that this information is follow-up information of a previously reported event.

All serious adverse events that occur during the study will be reviewed by Viralytics Medical Monitor, PPD
PPD

Report SAEs within 24 hours of discovery to:

- **Fax Number:** PPD
- **Precision Oncology Drug Safety email:** PPD

9.7. Regulatory Reporting Requirements

9.7.1 Annual Report

An annual report which includes the Development Safety Update Report (DSUR; listing, for example, all SARs and SUSARs) will be submitted to the US FDA, and any other applicable

regulatory authorities. The Sponsor is responsible for preparation and submission of the annual report.

9.7.2 Expedited Reporting

The Sponsor, or Sponsor's authorized representative or designee, is responsible for informing the FDA and the main REC of safety issues. For the FDA reporting the timelines for reporting are as follows:

- Fatal or life threatening SUSARs will be reported to the agency no later than **7 calendar days**, and
- All other SUSARs will be reported no to the agency later than **15 calendar days**.

9.8. Follow-up Procedures

After initially recording an AE or recording and reporting an SAE, the Investigator is required to follow each participant until resolution. Follow up information on an SAE should be reported to the Sponsor or designee.

Unless otherwise stated in the protocol, AEs and SAEs should be followed up until resolution or death of the trial subjects.

10. Trial Management and Oversight

10.1. Data Monitoring Committee

This study will utilize an independent Data Monitoring Committee (DMC). Per the DMC Charter, the DMC will meet for the following circumstances:

1. If there are 2 or fewer confirmed responses in the first 12 subjects within the first 12 months of starting treatment, as confirmed by the DMC, the study will be stopped due to futility of treatment.
2. If a Grade 3 or greater, CVA21 related DLT (except lymphopenia) is observed in 2 subjects among the first 6 treated.
3. At the discretion of the medical monitoring team or if overall DLT rate exceeds 30% of treated subjects.

The Medical Monitor will also review safety data after every 6 subjects have been treated and followed to at least Day 92. The DMC will review any findings to determine whether enrollment should be continued from a safety perspective.

The DMC will continue until the database is locked prior to preparation of the clinical study report.

10.2. Inspection of Records/Source Data

All case report forms will undergo quality assurance review by the Sponsor or delegate). All quality assurance reviews will include verification of the accuracy and integrity of data entered to case report forms. Incorrect data identified will be corrected by the site. The existence of adequate source documents for all data will be verified. All annual reports (continuing reviews) or publications will be submitted to the IRB for review and approval.

10.3. Monitoring

Study monitoring activities (Quality Control Reviews) are performed by a Contract Research Organization (CRO) or clinical research staff members who have completed specialized training in study monitoring procedures and human subject protections. Individuals who perform study monitoring activities do not report to Principal Investigators or research scientists and may not monitor studies for which they have direct responsibility.

Study monitoring activities are conducted regularly and include (but are not limited to) review and verification of the following:

- Eligibility
- Informed Consent process
- Adherence to protocol treatment plan
- Case Report Forms (CRFs)
- Source Documentation
- Adverse Events
- Regulatory Reporting

Results of study monitoring activities will be reported to applicable study personnel.

10.4. Risk Management

10.4.1 Potential Risks

There are three main potential risks:

- The most common potential risk for subjects in this study is an adverse event of CVA21-related “flu-like” illness. This is usually mild-moderate in severity and usually resolves within a few days.
- Signs and symptoms of transient septicemia such as fever, chills, rigors and tachycardia
- Although not observed in current studies, there is also a possible risk of CVA21 transmission to healthcare workers associated with patient care and conducting study assessments, and close contacts of the subjects themselves.

Should a case of CVA21 transmission be suspected, details should be recorded per the procedures outlined in **Appendix 15.9** and **15.11**.

Further information on potential risks and guidance for the Investigator may be found in the current CVA21 Investigator's Brochure.

10.4.2 Minimizing Risks

The risks identified in **Section 10.4.1** may be minimized as follows:

- While this risk is unlikely to be minimized, subject's symptoms may be addressed through the administration of antipyretics and non-steroidal anti-inflammatory drugs.
- Inadvertent transmission of CVA21 from treated subjects to healthcare workers can be minimized if procedures used in the handling of Biosafety Level 2 agents are followed. Viral administration should be performed in a manner to minimize potential exposure to other cancer suffering or immunocompromised individuals. Furthermore, systematic decontamination of surfaces that may directly come into contact with CVA21 or excretion samples must be undertaken, with an appropriate anti-viral agent (e.g., Virkon®, a sodium hypochlorite solution [5-6%] or formaldehyde [3%]). All materials utilized in the viral administration must be disposed of in appropriate infectious waste containers.

Any mishap such as accidental spillage or inoculation might expose a staff member to virus in ways not occurring naturally, although there is nothing to suggest they would be at particular risk as a result. Nevertheless, because of the above, it would be advisable that handling and inoculation of virus be done by staff trained in handling infectious agents and disposal of potentially infectious waste. Accidental spills from samples and operations should be adsorbed with paper tissues soaked with an appropriate anti-viral agent (e.g., Virkon®, a sodium hypochlorite solution [5-6%] or formaldehyde [3%]) and then with water. Tissues should then be discarded in proper containers designated for infectious laboratory/hospital waste. Please refer to the CVA21 Material Safety Data Sheet.

Inadvertent transmission of CVA21 from treated subjects to close contacts may be minimized through thorough patient education of potential transmission routes. Precautions are highlighted in a specific subject information sheet "What to do on a CAVATAK study".

11. Good Clinical Practice

11.1. Ethical Conduct of the Study

The study will be conducted in accordance with local laws and the principles of good clinical practice (GCP) and research for human subjects (Declaration of Helsinki).

A favorable ethical opinion will be obtained from the appropriate REC prior to commencement of the study.

11.2. Regulatory Compliance

The study will not commence until a Clinical Trial Authorization (CTA) is obtained from the appropriate Regulatory Authority. The protocol and study conduct will comply with the Medicines for Human Use (Clinical Trials) Regulations 2004, and any relevant amendments.

11.3. Investigator Responsibilities

The Investigator is responsible for the overall conduct of the study at the site and compliance with the protocol and any protocol amendments. In accordance with the principles of GCP, the following areas listed in this section are also the responsibility of the Investigator. Responsibilities may be delegated to an appropriate member of study site staff. Delegated tasks must be documented on a Delegation Log and signed by all those named on the list.

11.3.1 Informed Consent

The Investigator is responsible for ensuring informed consent is obtained before any protocol specific procedures are carried out. The decision of a participant to participate in clinical research is voluntary and should be based on a clear understanding of what is involved.

Participants must receive adequate oral and written information – appropriate Participant Information and Informed Consent Forms will be provided. The oral explanation to the participant should be performed by the Investigator or designated person, and must cover all the elements specified in the Participant Information Sheet/Informed Consent.

The participant must be given every opportunity to clarify any points they do not understand and, if necessary, ask for more information. The participant must be given sufficient time to consider the information provided. It should be emphasized that the participant may withdraw their consent to participate at any time without loss of benefits to which they otherwise would be entitled.

The participant should be informed and agree to their medical records being inspected by regulatory authorities but understand that inspection is undertaken by authorized personnel and their data will remain confidential.

The Investigator or delegated member of the trial team and the participant should sign and date the Informed Consent Form(s) to confirm that consent has been obtained. The participant should receive a copy of this document and a copy should be filed in the Trial Master File (TMF) or Investigator Site File (ISF) as appropriate.

11.3.2 Study Site Staff

The Investigator must be familiar with the IMP, protocol and the study requirements. It is the Investigator's responsibility to ensure that all staff assisting with the study are adequately informed about the IMP, protocol and their trial related duties.

11.3.3 Data Recording

The Investigator is responsible for the quality of the data recorded in the eCRF. Data should be entered within 72 hours of the subject's visit.

11.3.4 Investigator Documentation

Prior to beginning the study, each Investigator will be asked to provide particular essential documents to the Sponsor, including but not limited to:

- An original signed Investigator's Declaration (as part of the Clinical Trial Agreement documents);
- A signed copy of the Investigator's responsibilities letter issued by the Sponsor.

The Investigator, with the agreement of the Sponsor, will ensure all other documents required for compliance with the principles of GCP are retained in a TMF and that appropriate documentation is available in local ISFs.

11.3.5 GCP Training

All study staff must hold evidence of appropriate GCP training or undergo GCP training. This should be updated as appropriate throughout the trial.

11.3.6 Confidentiality

All laboratory specimens, evaluation forms, reports, and other records must be identified in a manner designed to maintain participant confidentiality. All records must be kept in a secure storage area with limited access. Clinical information will not be released without the written permission of the participant, except as necessary for monitoring and auditing by the Sponsor, its designee, Regulatory Authorities, or the REC. The Investigator and study site staff involved with this study may not disclose or use for any purpose other than performance of the study, any data, record, or other unpublished, confidential information disclosed to those individuals for the purpose of the study. Prior written agreement from the Sponsor or its designee must be obtained for the disclosure of any said confidential information to other parties.

11.3.7 Data Protection

All Investigators and study site staff involved with this study must comply with the requirements of the Data Protection Act 1998 with regard to the collection, storage, processing and disclosure of personal information and will uphold the Act's core principles. Access to collated participant data will be restricted to those clinicians treating the participants.

Computers used to collate the data will have limited access measures via assigned user names and passwords.

Published results will not contain any personal data that could allow identification of individual participants.

12. Study Conduct Responsibilities

12.1. Protocol Amendments

All modifications or amendments to the protocol or informed consent document must be approved by Viralytics before being submitted to the Institutional Review Board (IRB) for review and approval. All modifications and amendments will be documented with a new version number

and date. All changes to the informed consent document will include the date of the revision on the form. All IRB-reportable protocol deviations must be reported to Viralytics Medical Monitor.

No changes will be implemented until IRB approval is obtained except when a potential threat to patient safety exists.

The IRB will be notified of any significant deviations from the approved protocol. Documentation of all IRB correspondence will be maintained in the central regulatory file.

12.2. Protocol Violations and Deviations

The Investigator should not implement any deviation from the protocol without agreement from Viralytics, and appropriate IRB/REC and Regulatory Authority approval except where necessary to eliminate an immediate hazard to trial participants.

In the event that an Investigator needs to deviate from the protocol, the nature of and reasons for the deviation should be recorded in the CRF. If this necessitates a subsequent protocol amendment, this should be submitted to the IRB/REC and Regulatory Authority for review and approval if appropriate.

12.3. Study Record Retention

According to 21 CFR 312.62(c), the investigator shall retain required records for a period of 2 years following the date a marketing application is approved for the drug for the indication for which it is being investigated. If no application is to be filed or if the application is not approved for such indication, the investigator shall retain these records until 2 years after the investigation is discontinued or the IND is withdrawn and the FDA is notified.

The investigator must retain protocols; amendments; IRB/IBC approvals; completed, signed, dated consent forms; subject source documents; case report forms; quality monitoring reports; drug accountability records; and all documents of any nature regarding the study or subjects enrolled. Records may be placed in long-term storage after the study is completed. The location of long-term storage will be secure and easily accessed for regulatory purposes.

12.4. End of Study/Study Termination by Sponsor

The end of study is defined as the last participant's last visit.

The Investigators and/or Viralytics have the right at any time to terminate the study for clinical or administrative reasons.

The end of the study will be reported to the REC and Regulatory Authority within 90 days, or 15 days if the study is terminated prematurely. The Investigators will inform participants and ensure that the appropriate follow up is arranged for all involved.

A summary report of the study will be provided to the REC and Regulatory Authority within one year of the end of the study. An end of study report should also be issued to the funders at the end of funding.

12.5. Audits and Inspections

The study may be subject to audit by the Sponsor or by regulatory authorities. If such an audit occurs, the investigator must agree to allow access to required subject records. By signing this

protocol, the investigator grants permission to personnel from the Sponsor, its representatives and appropriate regulatory authorities for on-site monitoring of all appropriate study documentation, as well as on-site review of the procedures employed in CRF generation, where clinically appropriate.

12.6. Continuation of Drug Following End of Study

There are no plans at this time to provide CVA21 or pembrolizumab to subjects following completion of their participation in this study.

13. Reporting, Publications and Notification of Results

13.1. Authorship Policy

Ownership of the data arising from this study resides with the Sponsor. On completion of the study, the study data will be analyzed and tabulated, and a clinical study report will be prepared.

13.2. Publication

As a multicenter trial, the Sponsor intends to publish clinical data from all centers participating in the investigation. A publication committee selected by the Sponsor will submit draft manuscripts to all participating investigators for their comments. In conformity with the uniform requirements for manuscripts submitted to biomedical journals published by the International Committee of Medical Journal Editors (Kassirer and Angell, 1991), investigators whose contribution consists solely in the collection of data will not be named individually as authors. Rather, those investigators will receive a collective authorship as the “CVA21 CAPRA Study Group” and will be identified in a footnote.

Individual investigators and/or their associates subsequently may publish additional findings of this study in scientific journals or present them at scientific meetings, provided that the Sponsor is given ample opportunity to review any proposed abstract, manuscript, or slide presentation prior to its submission. This review is required to ensure that the Sponsor is aware of all written and oral presentations of the data and does not imply any editorial review or restriction of the contents of the presentation or use.

14. References

1. Siegel R, DeSantis MPH, Jemal A. Colorectal cancer statistics, 2014. CA Cancer J Clin 2014; 64:104-117.
2. Balch CM, Gershenwald JE, Soong SJ, Thompson JF, Atkins MB et al. Final version of 2009 AJCC melanoma staging and classification. J Clin Oncol. 2009 Dec 20; 27(36):6199-206.
3. Hodi FS, O'Day SJ, McDermott DF, Weber RW, Sosman JA et al. Improved survival with ipilimumab in Subjects with metastatic melanoma. N Engl J Med. 2010 Aug 19;363(8): 711-23.
4. Robert C, Thomas L, Bondarenko I, O'Day S, M D JW et al. Ipilumumab plus dacarbazine for previously untreated metastatic melanoma. N Engl J Med. 2011 Jun 30;364(26):2517-26.
5. Wolchok JD, Kluger H, Callahan MK, Postow MA, Rizvi NA et al. Nivolumab plus ipilimumab in advanced melanoma. N Engl J Med. 2013 Jul 11;369(2):122-33.
6. Sosman JA, Kim KB, Schuchter L, Gonzalez R, Pavlick AC et al. Survival in BRAF V600- mutant advanced melanoma treated with vemurafenib. N Engl J Med. 2012 Feb 23;366(8):707-14.
7. Chapman PB, Hauschild A, Robert C, Haanen JB, Asciento P et al. Improved survival with vemurafenib in melanoma with BRAF V600E mutation. N Engl J Med. 2011 Jun 30;364(26):2507-16.
8. Long GV, Trefzer U, Davies MA, Kefford RF, Asciento PA et al. Dabrafenib in Subjects with Val600Glu or Val600Lys BRAF-mutant melanoma metastatic to the brain (BREAK-MB): a multicentre, open-label, phase 2 trial. Lancet Oncol. 2012 Nov;13(11):1087-95.
9. Flaherty KT, Puzanov I, Kim KB, Ribas A, McArthur GA et al. Inhibition of mutated, activated BRAF in metastatic melanoma. N Engl J Med. 2010 Aug 26;363(9):809-19.
10. Jeffrey S. Weber, Ragini Reiney Kudchadkar, Bin Yu, Donna Gallenstein, Xiuhua Zhao, Alberto J. Martinez, Wenshi Wang, Geoffrey Gibney, Jodi Kroeger, Cabell Eysmans, Amod A. Sarnaik, and Y. Ann Chen. Safety, efficacy, and biomarkers of nivolumab with vaccine in ipilimumab-refractory or -naive melanoma. J Clin Oncol. 2013 Dec 1;31(34):4311-8
11. Hamid O, Robert C, Daud A, Hodi FS, Hwu WJ, Kefford R, Wolchok JD, Hersey P, Joseph RW, Weber JS, Dronca R, Gangadhar TC, Patnaik A, Zarour H, Joshua AM, Gergich K, Elassaiss-Schaap J, Algazi A, Mateus C, Boasberg P, Tumeh PC, Chmielowski B, Ebbinghaus SW, Li XN, Kang SP, Ribas A. Safety and tumor responses with lambrolizumab (anti-PD-1) in melanoma. N Engl J Med. 2013 Jul 11;369(2):134-44.
12. Robert C, Ribas A, Wolchok JD, Hodi FS, Hamid O, Kefford R, Weber JS, Joshua AM, Hwu WJ, Gangadhar TC, Patnaik A, Dronca R, Zarour H, Joseph RW, Boasberg P, Chmielowski B, Mateus C, Postow MA, Gergich K, Elassaiss-Schaap J, Li XN, Iannone R, Ebbinghaus SW, Kang SP, Daud A. Anti-programmed-death-receptor-1 treatment with pembrolizumab in ipilimumab-refractory advanced melanoma: a randomised dose-comparison cohort of a phase 1 trial. Lancet. 2014 Sep 20;384(9948):1109-17
13. Antoni Ribas, F. Stephen Hodi, Richard Kefford, Omid Hamid, Adil Daud, Jedd D. Wolchok, Wen-Jen Hwu, Tara C. Gangadhar, Amita Patnaik, Anthony M. Joshua, Peter Hersey, JeffreyS. Weber, Roxana Stefania Dronca, Hassane M. Zarour, Kevin Gergich, Xiaoyun (Nicole) Li, Robert Iannone, Soonmo Peter Kang, Scot Ebbinghaus, Caroline Robert. Efficacy and safety of the anti-PD-1 monoclonal antibody MK-3475 in 411 Subjects (pts) with melanoma. J Clin Oncol 32:5s, 2014 (suppl; abstr LBA9000^)
14. Spickard A, Evans H, Knight V, Johnson K. Acute respiratory disease in normal volunteers associated with Coxscackie A-21 viral infection. J Clin Invest. 1963;42:840,-853.
15. Couch RB, Cate TR, Douglas RG Jr, Gerone PJ, Knight V. Effect of route of inoculation on experimental respiratory viral disease in volunteers and evidence for airborne transmission. Bacteriol Rev. 1966;30:517-19.

16. Magee WE, Miller OV. Individual variability in antibody response of human volunteers to infection of the upper respiratory tract by coxsackie A21 virus. J Infect Dis. 1970;122:127-38.
17. Schiff GM, Sherwood JR. Clinical activity of pleconaril in an experimentally induced Coxsackievirus A21 respiratory infection. J Infect Dis. 2000 Jan; 181(1):20-26.
18. Shafren DR, Au GG, Nguyen T, Newcombe NG, Haley ES et al. Clin Cancer Res. 2004 Jan 1; 10(1 Pt 1):53-60.
19. Berry LJ, Au GG, Barry RD, Shafren DR. Potent oncolytic activity of human enterovirus, Coxsackievirus A21. Prostate. 2008 May 1;68(6): 577-87.
20. Skelding KA, Barry RD, Shafren DR. Systemic targeting of metastatic human breast tumor xenografts by Coxsackievirus A21. Breast Cancer Res Treat. 2009 Jan;113(1):21-30.
21. Au GG, Lincz LF, Enno A, Shafren DR. Oncolytic Coxsackievirus A21 as a novel therapy for multiple myeloma. Br J Haematol. 2007 Apr; 137(2):133-41.
22. Galanis E, Markovic SN, Suman VJ, Nuovo GJ, Vile RG et al. Phase II trial of intravenous administration of Reolysin® (Reovirus serotype-3-dearing strain) in Subjects with metastatic melanoma. Mol Ther. 2012; 20:1998-2003.
23. Hwang TH, Moon A, Burke J, Ribas A, Stephenson J et al. A mechanistic proof-of-concept trial with JX-594, a targeted multi-mechanistic oncolytic poxvirus, in Subjects with metastatic melanoma. Mol Ther. 2011;19(10):1913-22.
24. Wallack MK, Sivanandham M, Balch CM et al. A phase III randomized, double-blind multiinstitutional trial of vaccine melanoma oncolysate-active specific immunotherapy for Subjects with stage II melanoma. Cancer. 1995;75(1):34-42.
25. Kaufman HL, Kim DW, DeRaffele G, et al. Local and distant immunity induced by intralesional vaccination with an oncolytic herpes virus encoding GM-CSF in Subjects with Stage IIIC and IV melanoma. Ann Sur Oncol. 2010 Mar;17(3):718-30.
26. Kaufman HH, Andtbacka RH, Collichio FA, Amatruda T, Senzer NN et al. Primary overall survival (OS) from OPTiM, a randomized phase III trial of talimogene laherparepvec (T-VEC) versus subcutaneous (SC) granulocyte-macrophage colony stimulating factor (GM-CSF) for the treatment (tx) of unresected stage IIIB/C and IV melanoma. J Clin Oncol 32:5s, 2014 (suppl; abstr 9008a).
27. Puzanov I, Milhem MM, Andtbacka RHI, Minor DR, Hamid O, Li A, Chastain M et al. Primary analysis of a phase 1b multicenter trial to evaluate safety and efficacy of talimogene laherparepvec (T-VEC) and ipilimumab (ipi) in previously untreated, unresected stage IIIB-IV melanoma. J Clin Oncol. 2014; 32:5s, (suppl; abstr 9029a).
28. Pennock GK, Waterfield W, Wolchok JD. Patient responses to ipilimumab, a novel immunopotentiator for metastatic melanoma: how different are these from conventional treatment responses? Am J Clin Oncol. 2012;35:606-11.35.
29. Shafren D, Quah M, Wong Y, Andtbacka R.H and Au G. Combination of a novel oncolytic immunotherapeutic agent, coxsackievirus A21 and PD-1 blockade significantly reduces tumor growth and improves survival. Annals of Oncology (2014) 25 (suppl_4): iv361-iv372. 10.1093/annonc/mdu34.
30. Hoos A, Eggermont AM, Janetzki S, Hodi FS, Ibrahim R et al. Improved endpoints for cancer immunotherapy trials. J Natl Cancer Inst. 2010;102:1388-97.
31. Wolchok JD, Hoos A, O'Day S. Guidelines for the evaluation of immune therapy activity in solid tumors: immune-related response criteria. Clin Cancer Res 2009; 15:7412-20.
32. Simon R. Optimal two-stage designs for phase II clinical trials. Control Clin Trials. 1989; 10:1-10.

15. Appendices

15.1. Schedule of Procedures

Study Days		Day																				
	Screening (<28 days)[15]	1	3	5	8	29	50	71	92	113	134	155	176	197	218	239	260	281	302	323	344	365, 449, 533, 617 701[16]	386, 407, 428, 470, 491, 512, 554, 575, 596, 638, 659, 680
Visit window			±1 day	±1 day	±1 day	±1 day	± 4 days	± 4 days	± 4 days	± 4 days	± 4 days	± 4 days	± 4 days	± 4 days	± 4 days	± 4 days	± 4 days	± 4 days	± 4 days	± 4 days	± 4 days	± 7 days	± 7 days
Inclusion/Exclusion	X																						
Medical history	X	X																					
Full physical exam[1]	X	X		✓																			
Brief physical exam[1]					X	X		X		X		X	X	X	X	X	X	X	X	X		X	
Adverse Event assessment[2]		X	X	X	X	X	X	X	X	X	X	X	X	X	X	X	X	X	X	X		X	
Prior/Concomitant Medications	X	X	X	X	X	X	X	X	X	X	X	X	X	X	X	X	X	X	X	X		X	
Vital Signs, weight[1]	X	X	X		X	X		X		X		X	X	X	X	X	X	X	X	X		X	
CVA21 administration[3]		X	X	X	X	X	X	X	X	X	X	X	X	X	X	X	X	X	X	X			
Pembrolizumab[4] administration					X	X	X	X	X	X	X	X	X	X	X	X	X	X	X	X	X	X	X
CBC, Diff, plt[1]	X	X	X		X	X	X	X		X		X	X	X	X	X	X	X	X	X		X	
Chemistry panel[1]	X	X	X		X	X	X	X		X		X	X	X	X	X	X	X	X	X		X	
LDH[1]	X					X			X				X				X					X	
TSH, and if abnormal then free T3 and freeT4[1,5]	X					X		X		X		X	X	X	X	X	X	X	X	X		X	
PT, PTT[1]	X													X				X				X	

Study Days		Day																					
	Screening (<28 days)[15]	1	3	5	8	29	50	71	92	113	134	155	176	197	218	239	260	281	302	323	344	365, 449, 533, 617 701[16]	386, 407, 428, 470, 491, 512, 554, 575, 596, 638, 659, 680
ACTH, Cortisol[1]	X							X						X				X				X	
HIV, hepatitis[1,7]	X																						
Immunologic monitoring[1,8]	X	X				X		X		X				X				X				X	
CVA21 neutralizing ab titer[1]		X				X						X	X	X	X	X	X	X	X	X		X	
Serum CVA21 levels[1]		X	X	X	X	X	X	X	X	X	X	X	X	X	X	X	X	X	X	X		X	
12-lead ECG[9]	X																						
CT chest/abd/pelvis including response assessment[10]	X						X			X				X		X[17]		X		X[17]		X	
QOL assessment[11]		X					X			X				X				X				X	
Brain MRI[12]	X																						
Pregnancy test[6]	X	X				X		X		X													
Tumor biopsy[13]	X				X					X													
Photography of visible lesions[14]	X						X			X				X				X				X	

1. Physical examination, vital signs, weight and laboratory samples must be completed before dosing with either CVA21 or pembrolizumab.
2. Research nurse, or where appropriate the clinical research coordinator, will meet with the subject as indicated during office visits and contact the subject by phone weekly during the treatment and follow- up period through day 92 to assess and grade toxicities.
3. CVA21 up to a dose of 3 x $10^8 TCID_{50}$ (about 4.5 x 10^6 $TCID_{50}$/kg for a 70-kg subject) in a maximum volume of 4.0 mL by intratumoral administration, maximum of 19 sets of injections per subject (Days 1, 3, 5, 8, 29, 50, 71, 92, 113, 134, 155, 176, 197, 218, 239, 260, 281, 302 and 323).
4. Pembrolizumab will be administered at 2 mg/kg to be given over 30 minutes starting on Day 8. On days where both pembrolizumab and CVA21 are given, pembrolizumab should be administered after the CVA21 injection.

5. TSH will be tested periodically and if abnormal, then free T3 and free T4 will be assessed until normal. All subjects must have adequate thyroid function and/or in supplement to maintain TSH, T3, T4 in the normal range before enrollment at baseline.
6. Female subjects only. A serum or urine beta-HCG is necessary for women of child-bearing potential, to be collected prior to administration of CVA21 and/or pembrolizumab.
7. HIV and hepatitis screening will only be performed if there is a clinical history of HIV or hepatitis exposure.
8. PBMC will be collected at baseline (Screening or Day 1) and prior to pembrolizumab administration for doses 2, 4 and 6 and at study termination. All other timepoints will be optional. Collection will include 8 green top and 2 red top tubes for PBMC and serum. All samples will be collected and processed in the Tumor Immunology Laboratory. Please refer to protocol section 6.3 for additional details.
9. 12-lead ECG should be performed prior to administration of CVA21 and/or pembrolizumab. Abnormal readings will be assessed for clinical significance.
10. Other imaging studies as clinically appropriate as determined by the treating physician (e.g., PET scan, ultrasound of soft tissue lesions). Other imaging to assess tumor response at the discretion of the investigator. Imaging should be performed prior to administration of CVA21 and/or pembrolizumab.
11. QOL assessment using the FACT-BRM questionnaire to be filled out by the subject and data gathered by the research nurse or study coordinator at the time points indicated.
12. Only required at Screening for subjects who are symptomatic. Repeat brain MRI as clinically indicated.
13. Optional tumor biopsy of non-index, non-injected lesions greater than 10 mm if the subject consents and a specimen can be obtained using physical exam or ultrasound to identify lesions, prior to administration of CVA21 and/or pembrolizumab. Archival tissue may be used in place of a fresh biopsy at Screening.
14. Pictures of visible lesions (optional) should be obtained prior to administration of CVA21 and/or pembrolizumab.
15. The Screening period begins when the informed consent form is signed and continues until the first dose of CVA21 is administered. Screening assessments should be performed no more than 28 days prior to study Day 1. Screening physical examination, vital signs and laboratory assessments can also be used for study Day 1 assessments without having the need to repeat these tests, if these screening assessments were conducted within 7 days of study Day 1.
16. Follow-up visits will occur every 12 weeks (+/- 1 week) until progression. Subjects who withdraw from the study will be followed by telephone for survival only, every 3 months for the 1st year, then every 6 months until study closure.
17. CT/MRI scans at Day 239 and Day 323 will only be required for subjects who have an overall objective response of PR or CR at the previous three scans. E.g., If a subject has a CT/MRI-confirmed PR or CR at each of Days 50, 113, and 197, then the CT/MRI scan will be repeated six weeks later, at Day 239. Similarly, if a subject has a CT/MRI-confirmed PR or CR at each of Days 113, 197, and 281, then the CT/MRI scan will be repeated six weeks later, at Day 323.

15.2. ECOG Performance Status

0	Fully active, able to carry on all pre-disease activities without restriction (Karnofsky 90-100)
1	Restricted in physically strenuous activity but ambulatory and able to carry out work of a light or sedentary nature. For example, light housework, office work (Karnofsky 70-80)
2	Ambulatory and capable of all self-care but unable to carry out any work activities. Up and about more than 50% of waking hours (Karnofsky 50-60)
3	Capable of only limited self-care, confined to bed or chair 50% or more of waking hours (Karnofsky<br>30-40)
4	Completely disabled. Cannot carry on any self-care. Totally confined to bed or chair (Karnofsky<br>10-20)
5	Death (Karnofsky 0)

15.3. KEYTRUDA™ (Pembrolizumab) Package Insert

https://www.merck.com/product/usa/pi_circulars/k/keytruda/keytruda_pi.pdf

15.4. CVA21 Intratumoral Injection Technique

Distribution Technique for Injecting CVA21 into the Selected Tumor

The technique has been devised to allow for a maximal amount of distribution of CVA21 throughout the tumor.

Multiple lesions will be injected in a dose hyper-fraction pattern, starting with the largest lesion(s) (2.0 mL injected into tumors > 25 mm, 1.0 mL into 15 - 25 mm; 0.5 mL into 5 < 15 mm) to a 4.0 mL maximum. Measure the length of each tumor to be injected and determine the volume of CVA21 to be injected into each tumor. Sum the volumes to be injected so as to calculate the total volume of CVA21 required for the administration. The maximum volume of CVA21 to be administered is 4.0 mL. Following initial injection with CVA21, any injected lesion that reduces in diameter to< 0.5 cm will be injected with 0.1 mL of CVA21 as per the stated treatment schedule until the lesion completely resolves. The required volume CVA21 is withdrawn into a syringe. Exchange the withdrawal needle for a new needle and distribute the diluted virus solution into the tumors as described below.

**Tumor diameter**	**Volume of CVA21**
>25 mm	2.0 mL
15 – 25 mm	1.0 mL
5 < 15 mm	0.5 mL
< 5 mm*	0.1 mL

* only for lesions previously treated with CVA21

The distribution of virus is important to maximize the number of cancer cells initially infected with CVA21 virus. Maximizing the number of cancer cells and regions throughout the tumor that are initially infected theoretically will increase the amount of cancer cells destroyed. It will also increase the amount of viral progeny produced by the tumor, and therefore increase the chance of ongoing viremia. Viremia is critical for the seeding of remote tumors, and therefore this injection technique was created to accomplish this, as well as to have a uniform technique that will be the guide for all the subjects treated.

The following guidelines should be followed regarding order of preference for lesions to be injected, if 4.0mLs is determined to be insufficient to inject all qualifying lesions:

1. Lesions injected at baseline, both index and non-index
2. Index lesions that were present at baseline but were not previously injected
3. New lesions not present at baseline that are ≥ 5mm for non-lymph node (LN) lesions and ≥ 15mm for LN (short axis diameter).

Any newly injected lesions should continue to be injected for the remainder of the study, subject to order of preference above and without exceeding the maximum 4mL dose.

A 25-gauge needle should be used. A syringe is used that will accommodate the volume of CVA21 required. The volume of CVA21 to be administered will be determined based on the diameter of the tumor to be injected. To load the syringe with CVA21, remove vial from individual carton and thaw at room temperature (18-25°C). Do not leave the vial at room temperature (RT) for longer than is necessary to thaw the contents. A lab coat, safety glasses, sterile gloves and mask should be worn while loading the syringe with CVA21. Gently mix the vial for 5 seconds and tear off the plastic top. Use a luer-lock syringe of appropriate volume and 21-gauge needle to draw up the required volume. Remove air bubbles. Remove the withdrawal needle and replace with a 25-gauge capped needle. Hold on ice until required (2-8°C). Administer within 3 hours from loading the syringe and distribute into the tumors as described below.

The injection will be in 9 regions within the tumor on each injection day. The regions do not overlap, and they are selected by using the following landmarks.

The distribution of the viral solution will be as follows for Day 1, the first injection:

1. The center of the tumor is estimated and marked.
2. Marks are made around the periphery of the tumor at 45 degree radiants.
3. The site of the needle insertion is between the center mark and the 270-degree radiant. It should be at the approximate midpoint. This is the first dose injection site.
4. The volume of distribution is divided by 10, and will be distributed into 9 zones within the tumor.

The target zone for injection is the area within the tumor adjacent to the radiant marks, estimated to be approximately within the outer 20 % “rim” of the tumor. This will result in 8 injections. These first 8 injections should be aimed to be deep to the midline plane of the tumor.

The final injection is made directly deep to the predicted center of the tumor, and on this first dose is aimed at a depth above the midline and comprises 20% of the injection volume.

This is summarized in the following diagrams

TOP VIEW

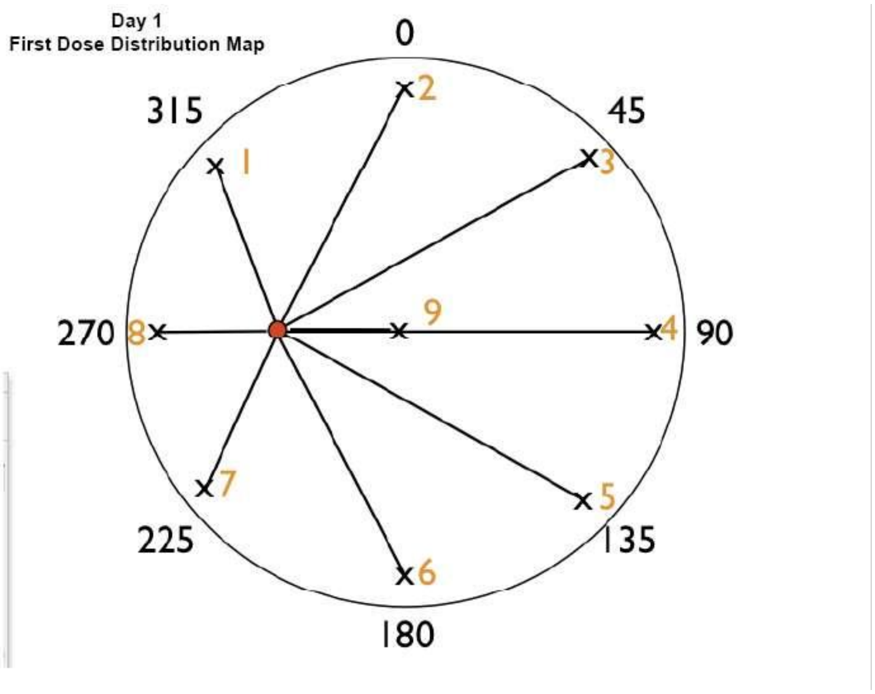

SIDE VIEW 1st injection

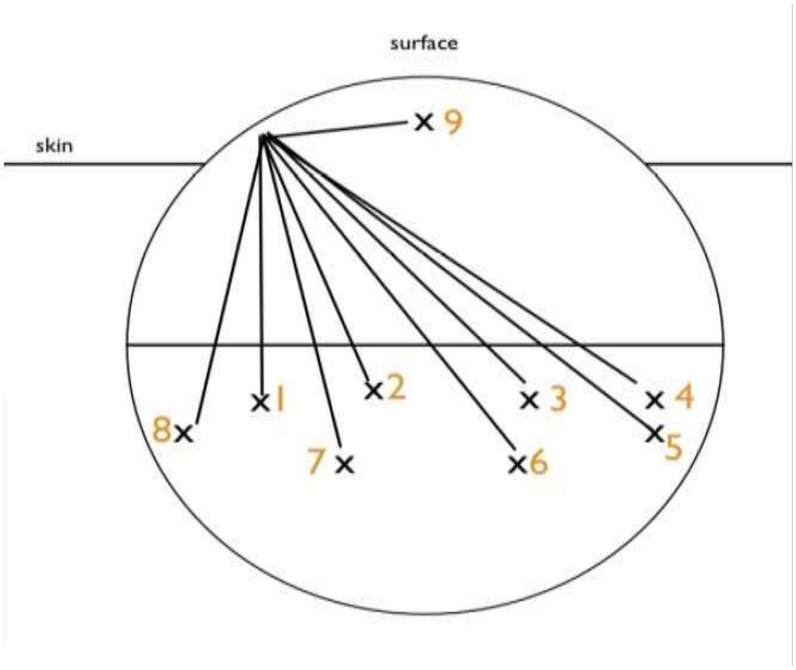
surface
skin
9
8
1
7
2
6
3
4
5

The second injection, on Day 3 (Visit 2) is as follows:

1. The injection area is to the right of the tumor, midway to the periphery in line with the 90-degree radiant.
2. The target injection zones are midway between the 45 degree radiants approximated to be within the 20% outer “rim” of the tumor. These injections should be made “superficial” to the mid-line depth of the tumor (as opposed to being deep to the mid-line depth of the tumor in the first day injection.
3. The final injection compromising 20% of the volume of distribution is targeted to the center of the tumor, deep to the mid-line, attempting to direct the solution into the outer 20% rim of the tumor.

The following diagrams demonstrate the injection target zones of the second dose of viral solution.

TOP VIEW

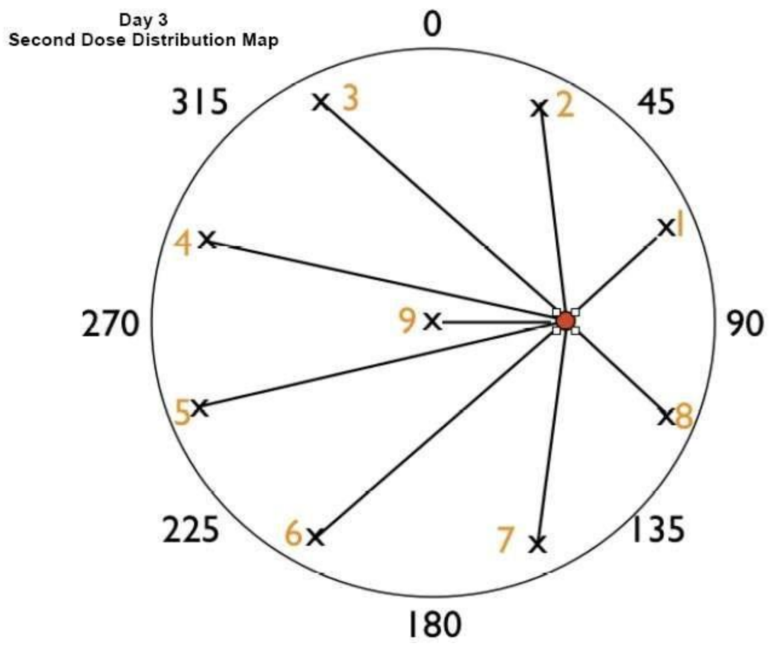

SIDE VIEW 2nd injection

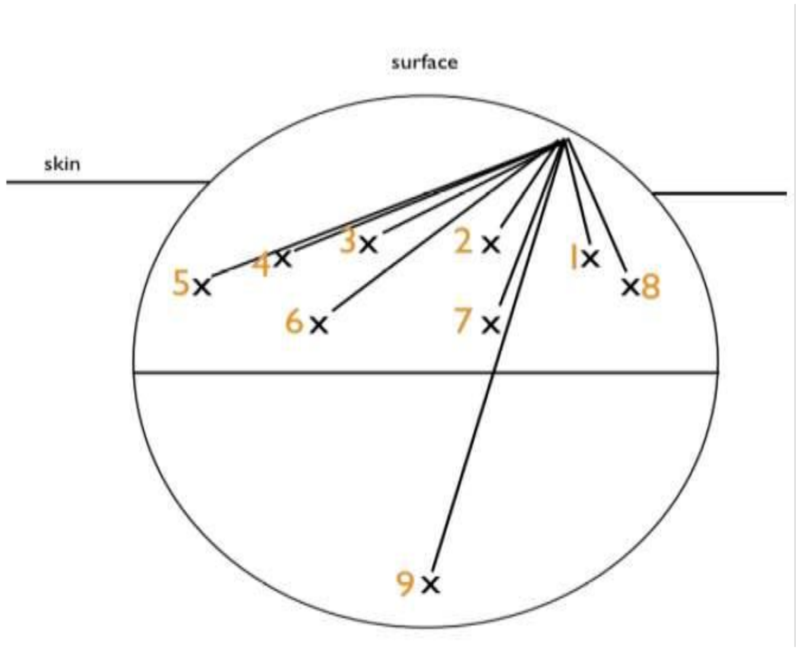

15.5. Listing of Auto-Immune Diseases

Subjects should be carefully questioned about any history of acquired immune deficiencies or autoimmune disease. Subjects are not eligible for the study if there is any history of immune deficiencies or autoimmune disease. Possible exceptions could be subjects with a medical history of atopic disease or childhood arthralgias where the clinical suspicion of an autoimmune process is low. In addition, transient autoimmune manifestations of an acute infectious disease that resolved upon treatment of the infectious agent are not excluded (e.g., acute Lyme arthritis). Vitiligo or immune- mediated hypothyroidism from prior melanoma immunotherapy are not excluded. Diseases that may be autoimmune-related include but are not limited to the following:

Acute disseminated encephalomyelitis

Addison's disease

Alopecia universalis

Ankylosing spondylitis

Antiphospholipid antibody syndrome Aplastic anemia

Asthma

Autoimmune hemolytic anemia

Autoimmune hepatitis

Autoimmune hypoparathyroidism

Autoimmune hypophysitis

Autoimmune myocarditis

Autoimmune oophoritis

Autoimmune orchitis

Autoimmune thrombocytopenic purpura

Behcet's disease

Bullous pemphigold

Celiac disease

Chronic fatigue syndrome

Chronic inflammatory demyelinating polyneuropathy

Churg-Strauss syndrome

Crohn's disease

Dermatomyositis

Dysautonomia

Eczema

Epidermolysis bullosa acquista

Gestational pemphigoid

Giant cell arteritis

Goodpasture's syndrome

Graves' disease

Guillain-Barré syndrome

Hashimoto's disease

IgA nephropathy

Inflammatory bowel disease

Interstitial cystitis

Kawasaki's disease

Lambert-Eaton myasthenia syndrome

Lupus erythematosus

Lyme disease – chronic

Meniere's syndrome

Mooren's ulcer

Morphea

Multiple sclerosis

Myasthenia gravis

Neuromyotonia

Optic neuritis

Opsoclonus myoclonus syndrome

Ord’s thyroiditis

Pemphigus

Pernicious anemia

Polyarteritis nodusa

Polyarthritis Polygrandular autoimmune syndrome

Primary biliary cirrhosis

Psoriasis

Reiter’s syndrome

Rheumatoid arthritis

Sarcoidosis

Scleroderma

Sjögren’s syndrome

Stiff-Person syndrome

Ulcerative colitis

15.6. Evaluation of Response

It is expected that clinically significant tumor regressions may be delayed, or occur after a period of progression in subjects who receive pembrolizumab-based immunotherapy. Response and progression will be evaluated in this study using immune-related response criteria (irRC) derived from modified WHO criteria as described by Hoos et al.[30, 31]

Measurable Disease: Measurability is defined as 5 × 5 mm or more on helical computer tomography scans. The sum of the perpendicular diameters (SPD) of index lesions at baseline is added to that of new lesions to calculate total tumor burden according to the following formula:

Tumor Burden = SPDindex lesions + SPDnew measurable lesions

Decrease in total measurable tumor burden is assessed relative to the baseline tumor burden, that is, SPD of all index lesions at baseline. The response categories irPD, irPR and irCR should be confirmed by a repeat consecutive assessment at least 4 weeks after the first assessment. Overall, immune-related response based on two or more tumor assessments is derived as shown in the following table:

Derivation of overall immune-related response for all assessed time points*			
**Measurable response**	**Non-measurable response**		**Overall response**
**Index and new measurable lesions (total measurable tumor burden)†**	**Non-index lesions**	**New measurable lesions**	**Using irRC**
100% decrease	Absent	Absent	irCR‡
≥ 50% decrease	Any	Any	irPR‡
< 50% decrease to < 25% increase	Any	Any	irSD
≥ 25% increase	Any	Any	irPD‡

* After Wolchok, et al (30). irCR = immune-related complete response – complete disappearance of all index and new measurable lesions; irPR = immune-related partial response – decrease in tumor volume ≥ 50% relative to baseline; irSD = immune-related stable disease – not meeting criteria for irCR or irPR, in absence of irPD; iPD = immune-related progressive disease – increase in tumor volume ≥ 25% relative to nadir.

† Index and non-index lesions are selected at baseline. Index lesions are measurable (> 5 x 5 mm), and non-index lesions are not measurable (< 5 x 5 mm, ascites, bone lesions, etc.). Changes are assessed relative to baseline and include measurable lesions only (> 5 x 5 mm).

‡ Assuming response and progression are confirmed by a second assessment at least 4 weeks apart. Note: as study visits after Day 106 occur 3 weeks apart, a second assessment 6 weeks later is preferred with the CT/MRI a study procedures all being conducted within the allowed study window.

Using irRC, the appearance of new lesions alone does not constitute irPD if they do not add to the tumor burden by at least 25%. Subjects with new lesions but an overall tumor burden decrease qualifying for partial response (≥ 50% decrease) or qualifying for stable disease (< 50%

decrease to > 25% increase) are considered to have irPR or irSD, respectively (same percentage changes including new lesions).

Subjects who develop isolated CNS progression but have extracranial disease control will be given the option to remain on protocol treatment while CNS-directed treatment (e.g., stereotactic radiosurgery or whole-brain radiotherapy) is added (if clinically indicated and feasible). Date of PFS will be recorded as date of CNS progression; however, if CNS control can be established, such subjects will be allowed to continue on protocol treatment until extra-CNS progression, with these additional data being captured in the trial

Note: irRC does not include guidelines for the assessment of response in lymph nodes when designated as index lesions. Therefore, for the purposes of this study, the rules used in RECIST1.1 will be used. Specifically:

1. Lymph nodes are considered normal structures when less than 10 mm in short axis diameter (SAD) and pathologically enlarged if >10 mm. To be considered an index lesion and measurable, a lymph node must be ≥ 15 x ≥ 15 mm in size.
2. The SPD for nodal index lesions is added to the SPD of all index lesions at Baseline and at subsequent response assessments.
3. Lymph nodes that reduce to ≤ 10 x 10 mm should be recorded but are not included in the SPD of all index lesions.
4. For non-target lesions, lymph nodes below less than 15 x 15 mm can be designated as non-target lesions if CT shows increased FDG uptake (SUV greater than or equal to 3).

15.7. Monitoring Plan, Quality Control and Quality Assurance

This study will be monitored at all stages of its development by the clinical research organization (CRO) designated by the Sponsor. Monitoring visits of active sites will occur every 4-8 weeks, with weekly telephone and/or email communication to assure that the clinical trial is conducted according to protocol and complies with guidelines of Good Clinical Practice. On-site review of eCRFs and Investigational Medicinal Product inventory and dispensing records will include a review of forms for completeness and clarity and consistency with source documents available for each subject. Note that a variety of original documents, data and records will be considered as source documents in this trial.

Medical advisors and clinical research associates or assistants may request to witness patient evaluations occurring as part of this protocol. The investigator and appropriate personnel will be periodically requested to attend meetings/workshops organized by the Sponsor to assure acceptable protocol execution. The study may be subject to audit by the Sponsor or by regulatory authorities. If such an audit occurs, the investigator must agree to allow access to required patient records. By signing this protocol, the investigator grants permission to personnel from the Sponsor, its representatives and appropriate regulatory authorities for on-site monitoring of all appropriate study documentation, as well as on-site review of the procedures employed in eCRF generation, where clinically appropriate.

15.8. Healthcare Worker Instructions in case of Suspected CVA21 Transmission

General Information

Coxsackievirus A21 is a naturally occurring virus that induces mild upper respiratory symptoms during natural infection of humans. CVA21 is acquired naturally by the respiratory route and circulates naturally in the community from time to time. Based on a number of small serology monitoring studies, around 20-50% of the general population possess pre-existing immunity to CVA21. CVA21 naturally infects cells in the respiratory tract that are known to express ICAM-1, thereby resulting in the development of “common cold”-like symptoms commonly observed during natural and experimental infection. During experimental human infection with CVA21 via intranasal administration, infectious virus has been recovered in nasal washing, throat swab, sputum and feces.

As such there is a possibility that subjects in the study may excrete virus from the respiratory or gastrointestinal tract for some days after CVA21 administration. As such, caregivers, family members, clinical trial staff and healthcare workers must be aware of this potential shedding and follow the guidance listed below. However, it must be noted that potential shedding is unlikely to be quantitatively different from a naturally acquired infection.

Specific Instructions

The Sponsor recommends that during the course of the study all staff involved in the viral administration and the collection of excretion samples wear protective gloves and face masks that are disposed of immediately. Although CVA21 is primarily transmitted via aerosols, injected lesions should be covered with an occlusive dressing that covers the entire lesion. This dressing should remain in place until the next scheduled visit, when it will be replaced by a new dressing. Viral administration should be performed in a manner to minimize potential exposure to other Subjects with cancer or immunocompromised individuals. Furthermore, systematic decontamination of surfaces that may directly come into contact with viral inoculum or excretion samples must be undertaken with an appropriate antiviral agent (e.g., Virkon®, a sodium hypochlorite solution [56%] or formaldehyde [3%]). All materials utilized in the viral administration must be disposed of in appropriate infectious waste containers.

In the event of an accidental spillage of CVA21, the healthcare should wear a protective laboratory gown, protective gloves and a face mask that are disposed of immediately following the decontamination process. The contaminated site should be immediately be covered with an appropriate antiviral agent (e.g., Virkon®, a sodium hypochlorite solution [5-6%] or formaldehyde [3%]) for at least 10 minutes and then adsorbent tissue/paper used to collect the antiviral solution. The contaminated site should then be thoroughly wiped with a clean tissue containing fresh antiviral solution and then wiped dry with fresh tissue. Contaminated tissues should be disposed of in appropriate infectious waste containers. If the contaminated area contains fragments of the vial containing the CVA21 solution, then they should be immediately placed in a sealed bio-hazard sharps container.

Any mishap such as accidental spillage or inoculation might expose a staff member to virus in ways not occurring naturally, although there is nothing to suggest they would be at particular risk as a result. Nevertheless, because of the above, it would be advisable that handling and

inoculation of virus be done by staff trained in handling infectious agents and disposal of potentially infectious waste.

In the event that a health care worker accidentally injects themselves with CVA21, encourage bleeding from the skin wound and wash the injured area with copious soapy water, disinfectant, scrub solution or water and cover the site of injection with an occlusive dressing.

If a health care worker experiences symptoms of a “common cold infection” following recent contact with a study subject, then the duration and severity of the symptoms should be noted on the supplied record form. Furthermore, a throat swab or sputum sample may be taken for analysis of the presence of CVA21.

15.9. Record of Illness by Health Care Worker

Name of health care worker	Age of contact	Sex Male or Female	Date of first contact with subject after treatment	Date of last contact with subject prior to developing illness	High temperature. Please record	Nausea (YES or NO)	Sore throat (YES or NO)	Diarrhea (YES or NO)	Duration of illness (number of days)	Throat swab of sputum sample taken (YES or NO)	Other comments about illness

15.10. Instructions for Close Contacts for CVA21 Transmission

General Information

Coxsackievirus A21 is a naturally occurring virus that induces mild upper respiratory symptoms during natural infection of humans. CVA21 is acquired naturally by the respiratory route and circulates naturally in the community from time to time. Based on a number of small serology monitoring studies, around 20-50% of the general population possesses pre-existing immunity to CVA21. CVA21 naturally infects cells in the respiratory tract that are known to express ICAM-1, thereby resulting in the development of "common cold"-like symptoms commonly observed during natural and experimental infection. During experimental human infection with CVA21 via intranasal administration, infectious virus has been recovered in nasal washing, throat swab, sputum and feces.

As such there is a possibility that subjects in the study may excrete virus from the respiratory or gastrointestinal tract for some days after CVA21 administration. As such, caregivers, family members, clinical trial staff and healthcare workers must be aware of this potential shedding and follow the guidance listed below. However, it must be noted that potential shedding is unlikely to be quantitatively different from naturally acquired infection.

Specific Instructions

Subjects in the study may excrete infectious virus in respiratory and gastrointestinal tract secretions for a number of weeks following the initial viral injection. Precautions that are normally undertaken during the general prevention of respiratory infections should be followed. Hands should be washed with soap and warm water immediately following handling of feces/fecal soiled clothing, and material exposed to respiratory secretions such as tissues and handkerchiefs.

Caregivers and family members should observe personal hygiene (i.e., hand washing) following contact with recently treated subjects. Caregivers who present symptoms of a cold or flu should present to a study investigator to provide a sample for analysis of the underlying infection.

If the occlusive dressing(s) on the study subject is accidentally removed, then a new occlusive dressing should be placed over the sites of injection. Old or soiled occlusive dressings should be placed in a sealed plastic bag and disposed in the house-hold rubbish container. Subjects will be sent home with extra dressings should they need new bandages.

If a close contact experiences symptoms of a "common cold infection" following recent contact with a study subject, then the duration and severity of the symptoms should be noted on the supplied record form and given to the healthcare workers of the site administering the CVA21 injections. If the close contact is concerned that they have contracted a CVA21 infection, a throat swab or sputum sample may be taken by the healthcare workers of the site administering the CVA21 injections for analysis of the presence of CVA21.

15.11. Record of Illness by Close Contact

Name of close contact	Age of contact	Sex Male or Female	Date of first contact with subject after treatment	Date of last contact with subject prior to developing illness	High temperature.<br><br>Please record	Nausea<br><br>(YES or NO)	Sore throat<br><br>(YES or NO)	Diarrhea<br><br>(YES or NO)	Duration of illness<br><br>(number of days)	Throat swab of sputum sample taken<br><br>(YES or NO)	Other comments about illness

15.12. FACT-BRM Questionnaire

Below is a list of statements that other people with your illness have said are important. Please circle or mark one number per line to indicate your response as it applies to the past 7 days.

	PHYSICAL WELL-BEING	Not at all	A little bit	Some-what	Quite a bit	Very much
GP1	I have a lack of energy	0	1	2	3	4
GP2	I have nausea	0	1	2	3	4
GP3	Because of my physical condition, I have trouble meeting the needs of my family	0	1	2	3	4
GP4	I have pain	0	1	2	3	4
GP5	I am bothered by side effects of treatment	0	1	2	3	4
GP6	I feel ill	0	1	2	3	4
GP7	I am forced to spend time in bed	0	1	2	3	4

	SOCIAL/FAMILY WELL-BEING	Not at all	A little bit	Some-what	Quite a bit	Very much
GS1	I feel close to my friends	0	1	2	3	4
GS2	I get emotional support from my family	0	1	2	3	4
GS3	I get support from my friends	0	1	2	3	4
GS4	My family has accepted my illness	0	1	2	3	4
GS5	I am satisfied with family communication about my illness	0	1	2	3	4
GS6	I feel close to my partner (or the person who is my main support)	0	1	2	3	4
Q1	Regardless of your current level of sexual activity, please answer the following question. If you prefer not to answer it, please mark this box and go to the next section.					

GS7	I am satisfied with my sex life	0	1	2	3	4

Please circle or mark one number per line to indicate your response as it applies to the past 7 days.

	EMOTIONAL WELL-BEING	Not at all	A little bit	Some-what	Quite a bit	Very much
GE1	I feel sad	0	1	2	3	4
GE2	I am satisfied with how I am coping with my illness	0	1	2	3	4
	I am losing hope in the fight against my illness	0	1	2	3	4
GE4	I feel nervous	0	1	2	3	4
GE5	I worry about dying	0	1	2	3	4
GE6	I worry that my condition will get worse	0	1	2	3	4

	FUNCTIONAL WELL-BEING	Not at all	A little bit	Some-what	Quite a bit	Very much
GF1	I am able to work (include work at home)	0	1	2	3	4
GF2	My work (include work at home) is fulfilling	0	1	2	3	4
GF3	I am able to enjoy life	0	1	2	3	4
GF4	I have accepted my illness	0	1	2	3	4
GF5	I am sleeping well	0	1	2	3	4
GF6	I am enjoying the things I usually do for fun	0	1	2	3	4
GF7	I am content with the quality of my life right now	0	1	2	3	4

Please circle or mark one number per line to indicate your response as it applies to the past 7 days.

	ADDITIONAL CONCERNS - Physical	Not at all	A little bit	Some-what	Quite a bit	Very much
BMT6	I get tired easily	0	1	2	3	4
HI12	I feel weak all over	0	1	2	3	4
C6	I have a good appetite	0	1	2	3	4
BRM1	I have pain in my joints	0	1	2	3	4
BRM2	I am bothered by the chills	0	1	2	3	4
BRM3	I am bothered by fevers (episodes of high body temperature)	0	1	2	3	4
BRM10	I am bothered by sweating	0	1	2	3	4

	ADDITIONAL CONCERNS - Mental	Not at all	A little bit	Some-what	Quite a bit	Very much
HI8	I have trouble concentrating	0	1	2	3	4
HI9	I have trouble remembering things	0	1	2	3	4
BRM7	I get depressed easily	0	1	2	3	4
BRM8	I get annoyed easily	0	1	2	3	4
BRM9	I have emotional ups and downs	0	1	2	3	4
HI6	I feel motivated to do things	0	1	2	3	4
